# Lightweight intrusion detection system using multiscale attention 1D CNN for large scale internet of things

**DOI:** 10.3389/frai.2026.1857306

**Published:** 2026-07-20

**Authors:** Dwarsala Sireesha, Kakelli Anil Kumar

**Affiliations:** School of Computer Science and Engineering, Vellore Institute of Technology, Vellore, India

**Keywords:** 1D-CNN, DDos, DOS, IDS, IoT, multiscale attention

## Abstract

The Internet of Things (IoT) and its applications are increasing rapidly over the years. Due to the wide variety of IoT applications, cyber attackers are exploring strong attacking methods and patterns to damage the IoT networks in real-time applications even if the IoT network is secure. To protect the IoT networks, it is essential to design and develop a real-time intrusion detection system that can detect the attacking patterns and methods and prevent them immediately. To achieve this goal, we have proposed an intrusion detection system using multiscale attention 1D convolutional neural networks for efficient detection of all major attacks. Our proposed mechanism integrates multi-scale convolutional kernels with a dual attention mechanism for computationally efficient intrusion detection. This mechanism has extracted spatial features to discriminate against the normal and malicious IoT traffic patterns. The experiment evaluation of the proposed work has tested two datasets, UNSW-NB15 and UM-NIDS 24, to evaluate its inference efficiency and intrusion detection capability. The proposed IDS has demonstrated the best performance in comparison to state-of-the-art models and achieved an accuracy of 91.03% on the UM-NIDS and an accuracy of 99.37% on the UNSW-NB15. Based on the experimental results and analysis, we can conclude that the proposed IDS with MA-1D-CNN is a lightweight, feature-efficient, and high-precision model for the real-time attack detection in large-scale IoT networks.

## 1 Introduction

**Figure 1:**
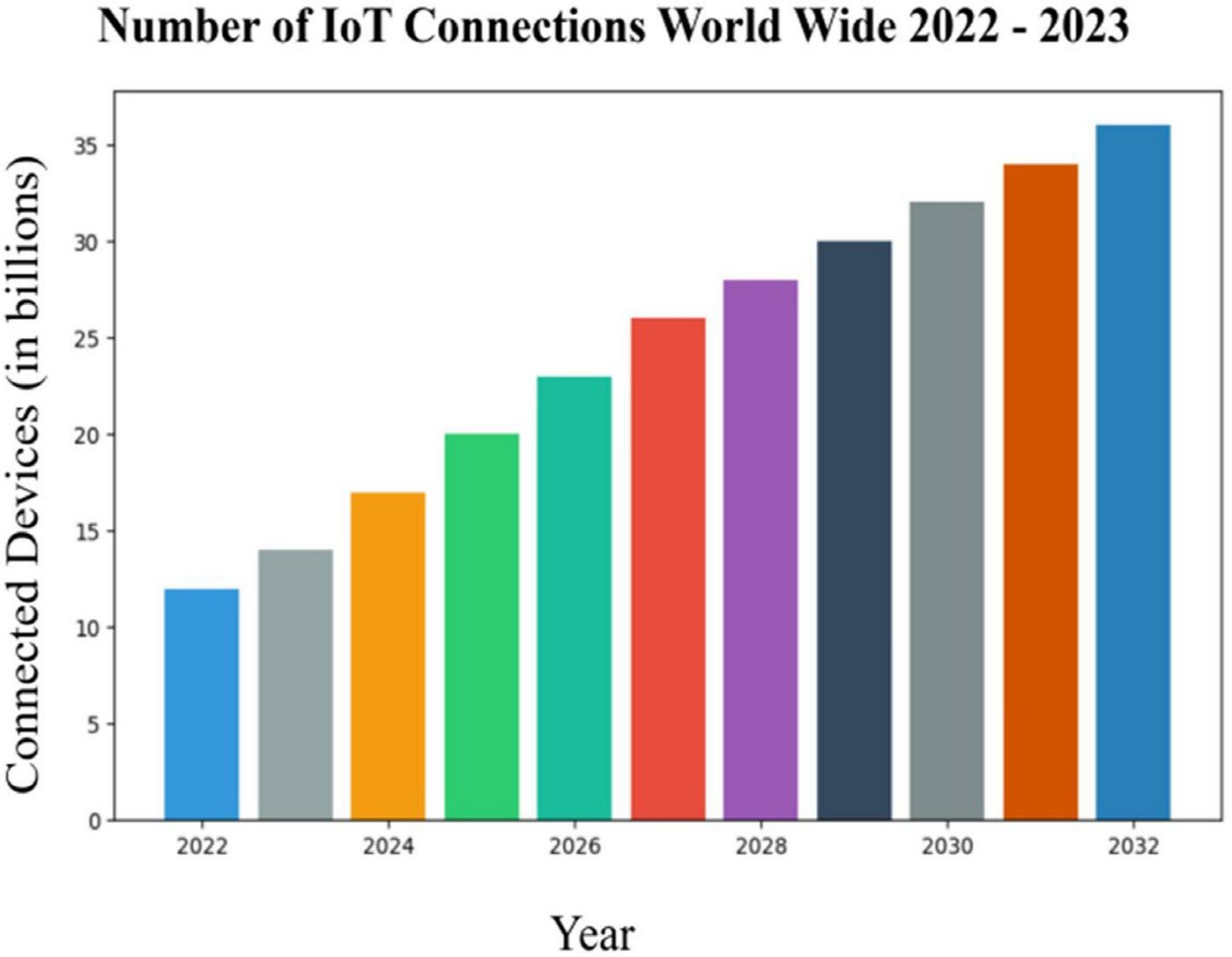
The growth and use of IoT devices statistics.

The IoT has been growing exponentially in recent years, which has transformed industries such as industrial automation, healthcare, transport, and intelligent urban environments through comprehensive solutions, connectivity, and real-time data exchange between billions of devices worldwide. Figure 1 shows that the number of networked IoT strategies worldwide is expected to surpass 30 billion by 2032, representing the rapid growth of the global IoT ecosystem. However, such hyper-connectivity has greatly expanded the attack surface, making IoT infrastructures much more vulnerable to cyberattacks, especially DoS and DDoS attacks, which take advantage of the constrained computation and security capabilities of IoT devices. A recent report showed that DDoS attacks will increase by 82% in 2024, driven primarily by IoT-based botnets.

Traditional IDSs, including signature-based and rule-based approaches, tend to fail in identifying evolving and dynamic attacks because of their static definitions, inability to detect zero-day exploits, and high false-positive rates. These loopholes call for the design of smart, adaptive, and scalable intrusion detection techniques that can efficiently handle large-scale, heterogeneous IoT environments. Because they can automatically extract high-level and discriminative features from high-dimensional network traffic data, deep-learning-based IDS models are quite successful. Among the various categories of deep learning models, CNNs and attention models have emerged as the leading paradigms in intrusion detection because of their strong feature extraction and representation learning capabilities.

Earlier research has shown the success of IDS algorithms that utilize CNN as well as attention mechanisms. Hossain (2025) proposed a scalable deep-learning IDS for IoT structures using the CIC IoT-DIAD 2024 dataset, where the 1D CNN yielded the best results (99.12% for multi-class and 99.53% for binary) compared with the LSTM, RNN, and MLP models. Zhao et al. (2023) designed a CNN-AttBiLSTM model with a self-attention mechanism for feature fusion, with accuracy of 95.67% on the CIC-IDS2017 data set and hence enhanced detection accuracy and spatiotemporal representation. Abu Bakar et al. (2024) proposed FTG-Net-E, a hierarchical ensemble GNN designed to detect DDoS attacks using flow-to-traffic graphs.

The model achieved an accuracy of 99.67 and 99.29% F1-score, showcasing the strength of graph-based traffic inspection. Shebl et al. (2024) proposed DCNN, an 11-layer deep convolutional network with 99.25% accuracy on the CICIoT2023 dataset, which performed both binary and multi-class intrusion detection with good results. Benka et al. (2025) compared DL and ML models for Industrial Control Systems (ICS) security and concluded that 1D CNN provided the optimal accuracy vs. inference speed trade-off (accuracy of 0.92, Achieved an F1-score of 0.91), and making it a top contender for real-time IDS deployment. Sun et al. (2025) proposed a Multimodal Spatiotemporal Collaborative Network, which applied cross-modal transformers and wavelet-based feature guidance to achieve 99.90% accuracy and 99.80% F1-score on the CIC-IDS2017 dataset, highlighting multimodal fusion’s robustness. Li et al. (2025) introduced Bal-IDS, a robust IDS, which incorporates an improved 1D CNN and BiGRU with self-attention and adaptive sampling. It achieved 99.96% accuracy and extremely low false-alarm rates, demonstrating its effectiveness against low-frequency IoT attacks.

These state-of-the-art studies demonstrate the efficiency of hybrid Deep Learning Models (DLM) in intrusion accurately detection and at scale. However, most previous studies suffer from limitations pertaining to the extraction of temporal features, balancing datasets, and generalizing across heterogeneous IoT networks, thus necessitating a robust and interpretable hybrid IDS model. To address these challenges, the current study proposes a Multiscale Attention-Based 1D-CNN model, which shows high performance in the efficient detection of DDoS and DoS attacks in both the UM-NIDS and UNSW-NB15 IoT network datasets.

**Figure 2:**
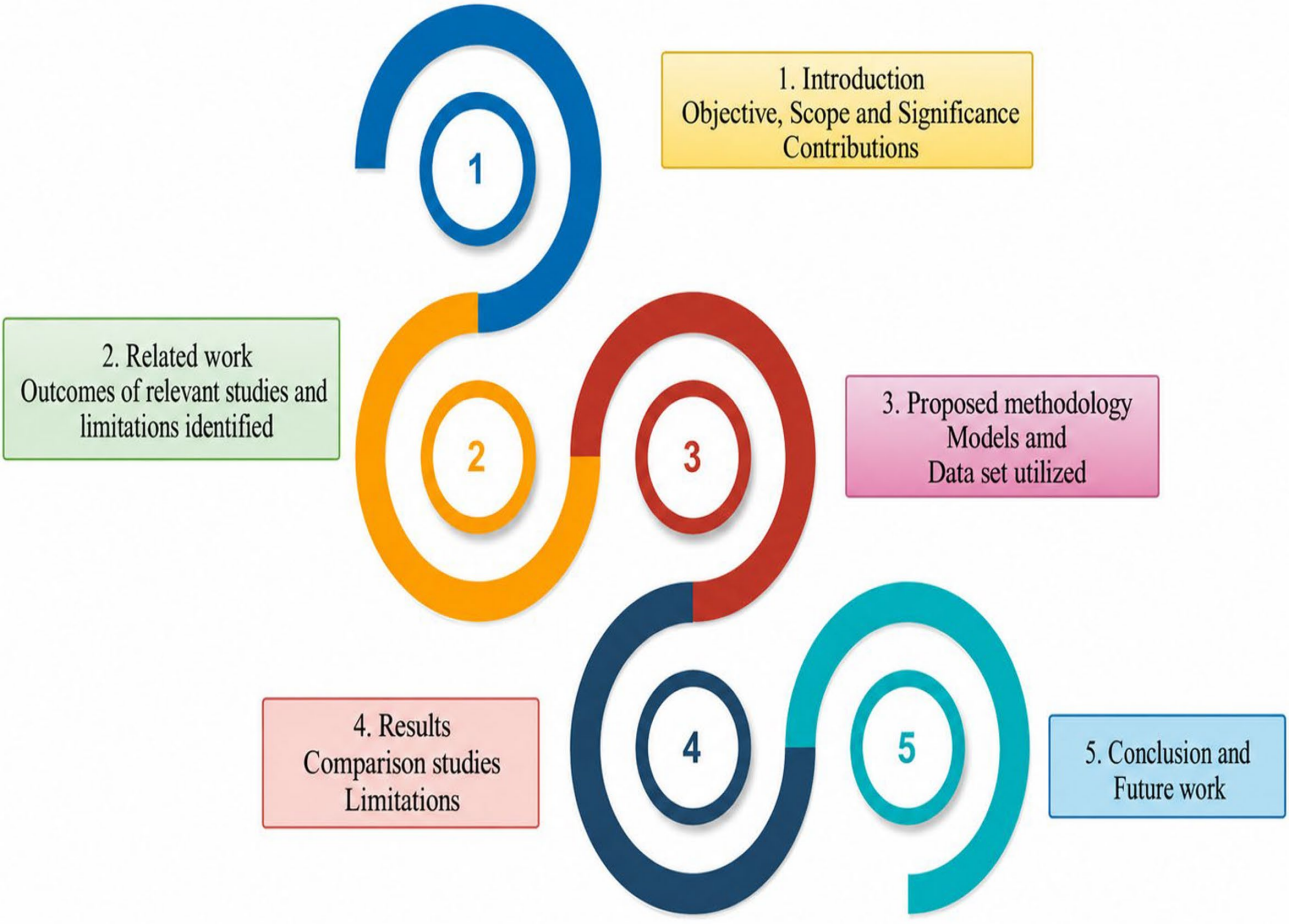
Organization of the proposed work.

**Figure 3:**
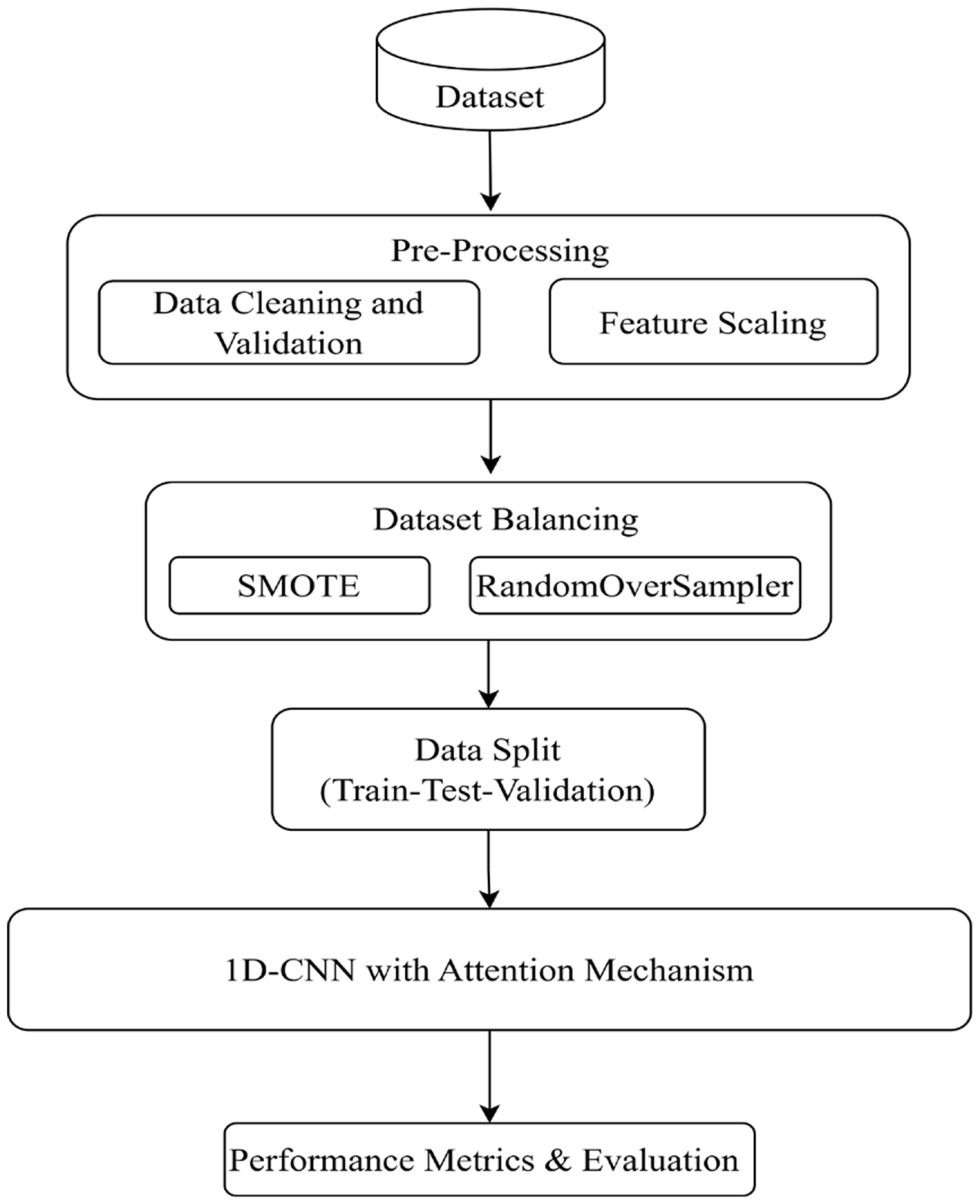
Workflow of 1D-CNN with Attention Mechanism.

The proposed workflow, illustrated in Figure 2, encompasses dataset balancing, preprocessing, train-test data splitting, hybrid model training, and evaluation on benchmark datasets. The basic design of the introduced framework, as illustrated in Figure 3, comprises a 1D-CNN for hierarchical temporal feature learning and an attention component for the adaptive highlighting of discriminating traffic features, thereby improving interpretability and achieving robust detection.

Table 1 shows the difference between the existing attention-based intrusion detection systems and the proposed MA-1D-CNN framework in terms of architecture. Traditional CNN-Attention models mostly use a single-scale convolution operation and a single attention mechanism, which restricts their capability to learn multiple level temporal dependency. CNN based BiLSTM-Attention models improve temporal feature extraction with the addition of recurrent layers at the cost of high computational complexity and inference latency. The IDS models based on CNN-CBAM, GNN, or Transformer enhance feature representation by leveraging sophisticated attention mechanisms, which generally demand high levels of computational resources and complexity in their design. The proposed MA-1D-CNN, however, combines multi-scale temporal feature extraction, dual attention refinement (Dense Attention and Squeeze-and-Excitation Attention), and residual learning in a light-weight and compact form. We are not aware of a single architecture that has all these modules and features simultaneously, making the proposed intrusion detection model architectural novel, in our knowledge.

**Table 1:** Comparative analysis of existing attention-based IDS architectures and the proposed MA-1D-CNN.

Architecture	Multi-scale feature extraction	Dual attention mechanism	Residual learning	Lightweight design
CNN-Attention (Zhao et al., 2023)	No	No	No	Yes
CNN-BiLSTM-Attention (Shieh et al., 2023)	No	No	No	No
CNN-CBAM-GRU (Hosseini et al., 2025)	Partial	Partial	No	No
Transformer-Based IDS (Harshdeep et al., 2025)	Yes	Yes	No	No
Proposed MA-1D-CNN	Yes	Yes	Yes	Yes

Here are the key findings from this study:

- A mixture deep model, which has a 1D-CNN for temporal feature learning and a method for giving attention to adaptively weight the discriminating features, was developed.
- Extensive empirical verification on two pieces of reference data, UNSW-NB15 and UM-NIDS, demonstrated the model’s scalability and generality for diverse IoT traffic patterns.
- Better detection performance: The proposed approach achieved 99.37% in terms of accuracy, recall, precision, and F1-score, surpassing the performance of other baseline DL-based and conventional ML approaches.

## 2 Related works

Deep learning-based IDS models have significantly evolved the architecture of CNNs, RNNs, and hybrids of both with attention mechanisms to outsmart adaptive cyber-attacks, such as DDoS and DoS attacks. Channel attention-based CNNs achieved the highest accuracy of 99.72% on the NSL-KDD dataset, emphasizing the role of attention in feature discrimination and classification. The contrast of CNN, RNN, and hybrid CNN–RNN attention mechanisms is noted, whereby attention-enabled RNNs significantly lower the test loss, whereas hybrid models are stumped in the balance of spatial and temporal feature extraction. Shieh et al. (2023) also validated the supremacy of 1D-CNNs by achieving improved classification performance against 14 classes of attacks in CICIDS2017 compared to traditional ANNs. Several IoT-focused IDS platforms have employed CNN and attention mechanisms to make them more resilient to dynamic threats. Al-Na’Amneh et al. (2024) introduced a hybrid SVM–CNN model that achieved 99% accuracy and F1-score when trained with CIC-IoT 2023, while (Kilichev and Kim, 2023) employed denoising autoencoders and ADASYN-based class balancing with a 1D-CNN, achieving good detection accuracy with high computational cost for NSL-KDD and UNSW-NB15. Wang and Ghaleb (2023) employed LSTM with attention and dimensionality reduction (PCA, UMAP), achieving binary accuracy of 99.09%. Yang et al. (2024) created a CNN–Attention IDS with minimal latency while maintaining high accuracy on the CSE-CIC IDS2018 dataset.

In IoT-related environments, a real-time hybrid IDS based on 1D-CNN, LSTM, RNN, and MLP with 99.53% accuracy on CIC-IoT-DIAD 2024, whereas Sasi et al. (2024) used a self-attention 1D-CNN–LSTM enhanced by SHAP-based feature selection with lower dimensionality without compromising accuracy. Singh et al. (2021) proposed Attention-Based CNNs (ABCNNs) applying mutual information for feature selection with satisfactory detection on Edge-IoTset, ToN-IoT, and CICIDS2017. Similarly, Padmavathy et al. (2024) introduced a biologically inspired 1D-CNN with better detection rates, and Huma et al. (2024) presented an inception-based spatial-attention CNN with an accuracy of 98.11%. In addition to deep learning, blockchain-based and hybrid approaches have been proposed to improve protocol awareness and the trustworthiness of data. Silivery et al. (2023) introduced a multi-class detection model that can classify DoS/DDoS subtypes at a fine-grained level. Zeeshan et al. (2022) presented a protocol-aware deep learning IDS that enhances cross-dataset generalization between Bot-IoT and UNSW-NB15 datasets. Alkadi et al. (2021) proposed a blockchain-based collaborative IDS that ensured data integrity with decentralization using consensus protocol. Despite these advances, class imbalance remains a significant problem, coupled with large computational requirements, inapplicability to encrypted traffic, and low flexibility for real-time IoT.

Alhassan (2025) introduced a Self-Adaptive Lightweight Attention Module (SLWAM)-based BiLSTM with spatial and channel attention integrated with a drift evaluation module, exhibiting high accuracy in CIC-DDoS2019 and adaptability to traffic dynamics. However, deploying these models on resource-constrained IoT nodes is Complicated. To address scalability, Shukla et al. (2025) proposed a distributed ensemble architecture on H2O.ai and Apache Storm, with more than 99% classification accuracy of 11 traffic classes in real time. Ensemble methods enhance scalability but at the cost of overhead in synchronization and integration in distributed frameworks. Hardware methods, such as those suggested by Sanlı (2024) utilized FPGA-based DoS detection with low response times in resource-constrained IoT systems but without agility against evolving attack patterns. Alslman et al. (2024) introduced denoising autoencoders (DAE) as a measure to defend IDS models against adversarial attacks, improving their robustness against white-box and black-box attacks. Similarly, Hussain et al. (2025) one group of researchers proposed an RL-based adaptive IDS for WSN with an accuracy of over 99% and minimal false positives. However, RL approaches require extensive training, which limits their real-time applicability. Optimization and evolutionary algorithms have also enhanced the reactivity of IDSs in industrial IoT (IIoT) environments. Alkhafaji et al. (2025) combined deep learning with Genetic Algorithms (GA) for feature selection with the best attributes, where accuracy on UNSW-NB15 was improved, though at a higher training expense. Srivastava and Sinha (2025b) used Frequent Pattern Growth (FP-Growth) with Jaccard similarity to identify DoS/DDoS, where accuracy was as high as 94.87% on CICIDS2017. Saidane et al. (2025) suggested that deep graph neural network models, D-GSAGE-MARC and GFN-GA, were proposed with multi-head attention and residual GAT layers. They achieved 99.97% accuracy and F1-score on the CIC-ToN-IoT and WUSTL-IIOT-2021 datasets.

Wen et al. (2025) introduced the Dynamic Weighted K-Asynchronous Federated Learning framework for federated intrusion detection, achieving 92, 91.3, and 85% accuracies on CICIDS2017and NSL-KDD UNSW-NB15, respectively with an emphasis on heterogeneous IoT devices. Prakash and Mohapatra (2025) proposed MA_BiRAE, which coupled adversarial training with a Multi-head Attention Bidirectional Residual Autoencoder, achieving 99.63 and 99.11% accuracy on the Aposemat IoT-23 and Bot-IoT datasets, respectively. Mohan et al. (2025) combined LSTM-FCNet and Gradient Boosting (GB) in a physical-layer security context and achieved 98.25% accuracy and an AUC of 0.99, which proved robustness under varying SNR conditions. Research on IDS in future networks has also been conducted. Harshdeep et al. (2025), a transformer-based 5G model, generated higher accuracy and efficiency than CNN- and RNN-based intrusion detection system (IDS) models. In vehicle environments, GC-LSTM-Ghost Net with CFACO optimization and rate limiting using RL ensured real-time DDoS protection (Jayakrishna and Prasanth, 2025). Similarly, AlexInception-BiLSTM-AttentionNet integrated with an SDN ensured an accuracy of over 99% for VANET intrusion detection in the same year. For IIoT, an Enhanced Firefly Algorithm (EFA)-optimized model augmented real-time detection with a class imbalance solution via SMOTE (Rahamathulla and Ramaiah, 2025). The CNN–CBAM–GRU hybrid model proposed by Hosseini et al. (2025) exhibited high detection accuracy in UNSW-NB15 and NSL-KDD, where the parallel architecture outperformed its sequential counterparts. Transformers have been applied to malware detection based on process utilization metrics (Natsos and Symeonidis, 2025), with satisfactory zero-day resilience and fewer false positives.

In Table 2, Traditional convolutional neural network attention-based IDS (CNN-attention IDS) structures adopt single-scale convolution or attention mechanisms, whereas the proposed MA-1D-CNN is designed with a single lightweight convolutional block (MA-1D) that combines the multiscale extraction of temporal features, dual attention refinement, and residual learning. The architecture enhances feature discrimination while keeping the computational efficiency in the various traffic mix of IoT use-cases.

**Table 2:** Architecture-level comparative analysis of IDS models.

Title of the paper	Architecture	Attention type	Multiscale	Residual	Feature efficiency technique	Limitation
CNN-AttBiLSTM Mechanism: A DDoS Attack Detection Method Based on Attention Mechanism and CNN-BiLSTM	CNN + BiLSTM + Self-Attention	Self-Attention	No	No	RF + Pearson Correlation Feature Selection	High computational complexity due to BiLSTM
Attention-Based Convolutional Neural Network for Intrusion Detection	CNN + Integrated Attention	Integrated Attention	No	No	Feature importance image arrangement	Limited temporal dependency modeling
Improved Intrusion Detection Method for IIoT using Attention Mechanisms, BiGRU, and Inception-CNN	Inception-CNN + BiGRU + Attention	Attention Mechanism	Yes	Partial	RF + Pearson + Hybrid Sampling	High training overhead
Efficient Self Attention-based 1D-CNN-LSTM for IoT Attack Detection	1D-CNN + LSTM + Self-Attention	Self-Attention	No	Yes	SHAP-based feature selection	Increased training latency
MA_BiRAE Malware Detection Technique	Deep RCNet + Residual Autoencoder	Multi-head Attention	Partial	Yes	IRF + RCNet feature extraction	Adversarial training complexity
Multimodal Spatiotemporal Collaborative Approach for Network Traffic Anomaly Detection	Multimodal Fusion + Wavelet + A5ttention	Feature Fusion Attention	Yes	No	Wavelet time-frequency extraction	Requires multimodal synchronization
Proposed MA-1D-CNN	Multiscale Attention-based 1D-CNN	Dense Attention + SE Attention	Yes	Yes	Nine-feature optimized lightweight representation	Requires hyperparameter tuning

Table 3 shows a critical comparison of some of the most prominent attention-based IDS reported in the latest literature. Prior works have shown that attention mechanisms, recurrent neural networks and graph-based architectures have been effective in enhancing intrusion detection performance. There are, however, a number of constraints. BiLSTM, LSTM, and GRU based architectures involve much computational overhead, and graph neural network architectures demand a lot of memory and processing power. Moreover, most models are either attention or temporal dependency learning but they have not simultaneously addressed multiscale feature extraction and residual learning. These limitations encouraged us to design the proposed MA-1D-CNN architecture which fused the multiscale convolutional feature extraction with Dense Attention, Squeeze-and-Excitation (SE) Attention and residual connections in one architecture. The integrated design will increase the ability to represent features and enhance computational efficiency, overcoming the limitations of current intrusion detection mechanisms.

**Table 3:** Critical comparison of representative attention-based intrusion detection systems and research gaps.

Study	Architecture	Key strength	Limitation
Li et al. (2025)	ADFCNN-BiLSTM + Multi-Head Attention	Combines deformable convolution, temporal learning, and attention mechanisms	High computational complexity due to BiLSTM and multi-head attention
Shieh et al. (2023)	1D-CNN	Effective feature extraction and multiclass attack classification	Limited temporal dependency modeling
Sasi et al. (2024)	Self-Attention 1D-CNN-LSTM	Integrates self-attention with explainable feature selection	Increased training latency caused by LSTM layers
Singh et al. (2021)	Attention-Based CNN (ABCNN)	Mutual information-based feature selection improves feature relevance	Lacks residual learning and multiscale feature extraction
Alhassan (2025)	SLWAM-BiLSTM	Adaptive spatial and channel attention for dynamic traffic patterns	Difficult deployment on resource-constrained IoT devices
Hosseini et al. (2025)	CNN-CBAM-GRU	Parallel attention architecture improves feature representation	Recurrent GRU layers increase computational overhead
Saidane et al. (2025)	D-GSAGE-MARC/GFN-GA	Graph neural networks with multi-head attention and residual learning	High memory consumption and computational requirements

## 3 Methodology

This methodology combines dataset preparation, preprocessing, feature scaling, class balancing, and the introduced CNN model into a cohesive pipeline. The data were initially gathered and cleaned and then subjected to adaptive sampling and normalization for statistical consistency. Class unevenness was overcome using SMOTE and oversampling to prevent bias. These pre-processed features are fed into the Multi-Scale Attention CNN, where several branches of convolution grasp the temporal relationships at varying scales, attention mechanisms spot features of interest, and SE blocks rescale channels. Residual connections and dense layers then project the extracted features into the intrusion classes (Normal, No Attack). This end-to-end flow guarantees robustness, scalability, and high detection accuracy for diverse datasets.

**Figure 4:**
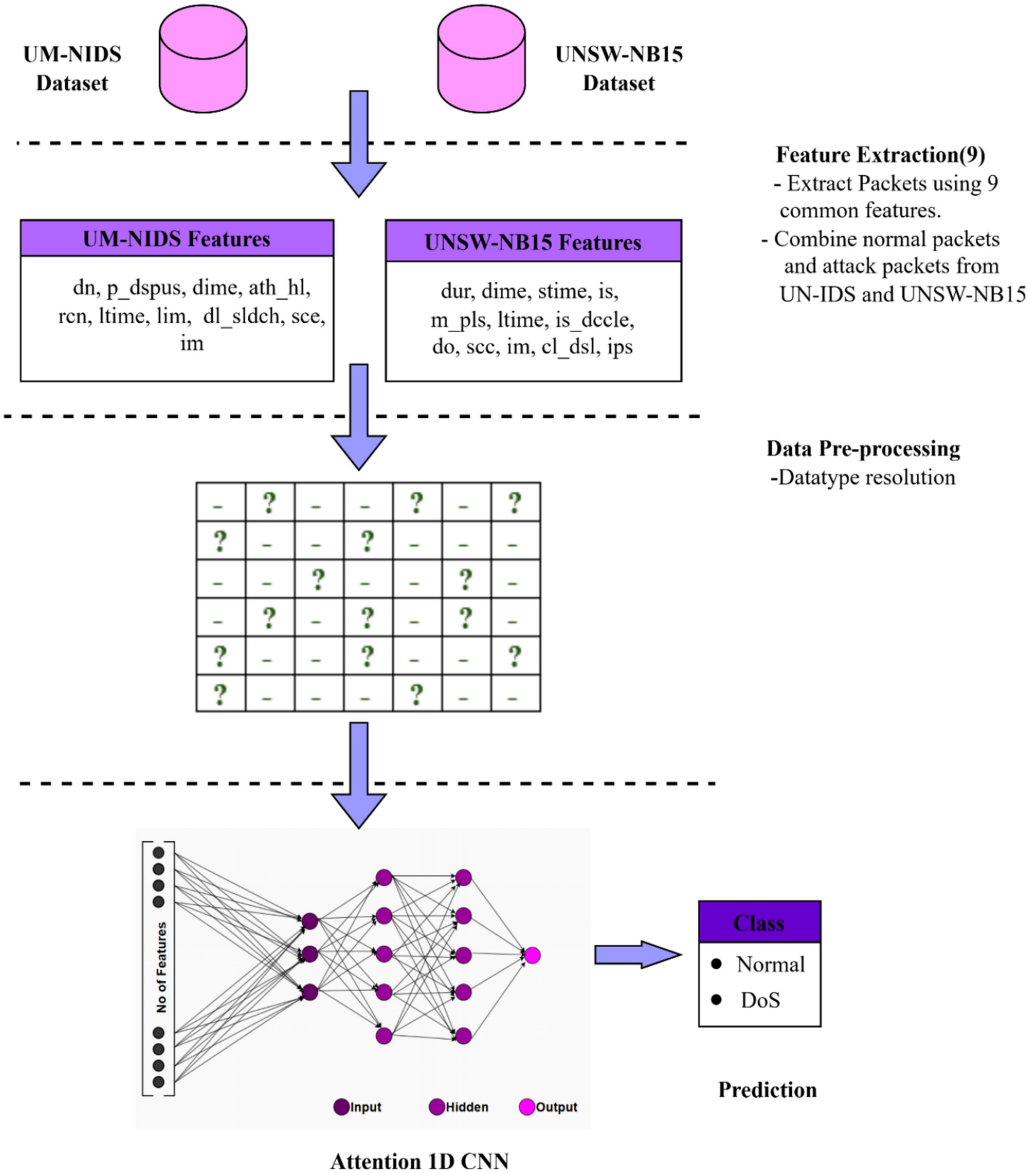
Architecture of the proposed multiscale attention-based 1D-CNN model for DoS attack detection in UMNIDS and UNSW-NB15 datasets.

Figure 4 depicts the procedure of the proposed Multiscale Attention-based 1D-CNN model designed to perform effective DDoS and DoS attack detection on the UMNIDS and UNSW-NB15 IoT network datasets. Records from network traffic were analysed from both datasets. Feature extraction is a process that identifies nine common features across datasets to offer compatibility in the event of aggregated assessment. Ordinary packets were selected from UMNIDS, and attack packets were obtained from both the UMNIDS and UNSW-NB15 datasets. Data preprocessing was conducted on the extracted features, including data type resolution and normalization, to ensure uniformity across heterogeneous sources. This enhances data consistency and makes the training more stable. Finally, the processed data were input into the self-attention-based 1D-CNN model. The network employs convolutional layers to learn hierarchical features and self-attention mechanisms to obtain inter-feature dependencies and improve the discrimination of subtle attack behaviors. Finally, the model classifies the traffic into Attack and Normal categories. The combined approach supports strong cross-dataset generalization and high accuracy in identifying DoS and DDoS attacks in IoT network environments.

### 3.1 Description of the dataset

This research was conducted in a dual-dataset comparison scenario using UM-NIDS and UNSW-NB15 datasets. The two real-world datasets allow for cross-dataset validation and valuable insights into model generalization capability of such intrusion detection model across different network systems and attack patterns.

### 3.2 UM-NIDS dataset overview

The UM-NIDS is envisioned as a next-generation,  unified, and standardized dataset to address the limitations of previous benchmark datasets. It combines several kinds of intrusion detection datasets into a same one structure with non-variable feature representation and sampling balance.

*Sampling strategy*: smart sampling with 100,000 representative samples was used.

*Feature space*: normalized and standardized homogeneous characteristic representation.

*Class distribution*: the class distribution of the native UM-NIDS dataset is very imbalanced with a few categories of attacks having less than 0.01% of the total attacks as shown in Table 4. Class balancing, described in Section 3.8, was used to mitigate class dominance and to enhance representation of minority attack classes during model training: undersampling the majority benign class and using SMOTE-based and random oversampling of the minority attack classes.

**Table 4:** Class distribution of UMNIDS datasets.

Class	Number of records	% of all the data
Benign (Normal)	25,165,295	33.12%
DDoS	3,046,028	4.01%
Reconnaissance	747,184	0.98%
Injection	72,534	0.10%
DoS	2,131,032	2.80%
Brute Force	382,215	0.50%
Password	15,277	0.02%
XSS	25,306	0.03%
Infiltration	161	<0.01%
Exploits	44,525	0.06%
Scanning	105,899	0.14%
Fuzzers	24,246	0.03%
Backdoor	19,126	0.03%
Bot	286,191	0.38%
Generic	215,481	0.28%
Analysis	2,677	<0.01%
Theft	1	<0.01%
Shellcode	1,511	<0.01%
MITM	174	<0.01%
Worms	174	<0.01%
Ransomware	149	<0.01%
Web Attacks	1,841	<0.01%
Total	75,987,976	100.00%

Format: Available in CSV format with a mix of numerical and categorical features.

Table 4 presents the traffic class distribution in the UM-NIDS dataset, which was used for both the testing and training of the proposed intrusion detection models. The benign/normal traffic accounted for 33.12% of the dataset, whereas DDoS and DoS were the dominant malicious traffic classes at 4.01 and 2.80%, respectively. For a few categories, such as Infiltration, Theft, Shellcode, MITM, and Ransomware, the distribution accounts for less than 0.01%. These results indicate a seriously imbalanced dataset. Class imbalance can adversely affect the model’s performance, especially when applied to minority attack classes. This requires preprocessing methods such as oversampling, undersampling, and synthetic data sampling (SMOTE). However, despite this imbalance, the UM-NIDS dataset allows for extensive coverage of modern cyberattacks. It can be tested as a binary (normal/attack) or multiclass problem. The dataset reflects realistic IoT traffic patterns, such as volumetric and behavioral attack characteristics of IoT traffic. Thus, it offers a valid point of reference for designing IDS models that can detect diverse types of attacks.

### 3.3 UNSW-NB15 dataset overview

The UNSW-NB15 dataset, developed by the ACCS, is a widely adopted benchmark dataset for intrusion detection research. It contains modern real-world network traffic with multiple attack vectors and realistic imbalances.

*Total records*: variable depending on the sampling arrangement.

*Attack types*: nine attack categories were considered along with normal traffic.

*Feature space*: rich temporal and network flow-based features.

*Class distribution*: realistic imbalance representing real-world network scenarios.

Table 5 shows the class allocation of the UNSW-NB15 dataset, which has approximately 2.54 million samples divided into 10 classes. The non-anomalous class was the largest, with 87.35%, and the high-level attack classes were generic (8.48%), exploits (1.75%), and DoS attacks were relatively low (0.64%). Some minority classes include backdoors, analysis, shellcode, and worms, which enrich the variety of the dataset. In the proposed method, class imbalance is considered in a multiscale attention-based 1D-CNN using attention-guided feature extraction and hierarchical convolutional filters. Stringent cross-validation with the datasets effectively verified the heterogeneous IoT network environments. The integration of both high-volume and sporadic attack instances within the datasets enables the training and testing of UNSW-NB15 for global DoS and DDoS detection. Its dense spatial and temporal patterns enable the model to capture discriminative features of different traffic behaviors. Overall, this dataset is a significant addition to the UM-NIDS for generalized intrusion detection testing.

**Table 5:** Class distribution of UNSW-NB15 dataset.

Class	Number of records	% of all the data
Non-anomalous (Normal)	2,218,761	87.35%
Generic	215,481	8.48%
Exploits	44,526	1.75%
Fuzzers	24,245	0.95%
DoS	16,354	0.64%
Reconnaissance	13,986	0.55%
Analysis	2,676	0.11%
Backdoor	2,330	0.10%
Shellcode	1,511	0.06%
Worms	174	0.01%
Total	2,540,044	100.00%

### 3.4 Comparative dataset properties

Table 6 presents a comparative impression of the datasets employed in this study, and Table 7 highlights the general traffic classes shared by the UMNIDS and UNSW-NB15 datasets. Each has normal (benign) traffic and multiple attack classes represented, including generic, exploit, fuzzers, DoS, and reconnaissance. Each has advanced malicious classes, such as Shellcode, Analysis, Backdoor, and Worms. The presence of these shared classes is indicative of cross-dataset occurrences and strengthens intrusion detection systems.

**Table 6:** Comparative overview of UM-NIDS and UNSW-NB15 datasets.

Property	UM-NIDS dataset	UNSW-NB15 dataset
Sampling strategy	Smart sampling (100,000 samples)	Full dataset with variable sampling
Feature space	Normalized, homogeneous features	Temporal and network flow-based features
Attack types	Multiple integrated categories	9 attack types + normal traffic
Class distribution	Original dataset highly imbalanced; balanced after SMOTE and oversampling	Imbalance reflects the real-world environment. Dataset is balanced after data preprocessing.
Format	CSV with numerical + categorical attributes	CSV with numerical attributes

**Table 7:** Common features between UMNIDS and UNSW-NB15.

Common class	Present in UMNIDS	Present in UNSW-NB15
Normal/Benign	Yes	Yes
Generic	Yes	Yes
Exploits	Yes	Yes
Fuzzers	Yes	Yes
DoS	Yes	Yes
Reconnaissance	Yes	Yes
Analysis	Yes	Yes
Backdoor	Yes	Yes
Shellcode	Yes	Yes
Worms	Yes	Yes

### 3.5 Data preprocessing pipeline

Intelligent Data Loading with Adaptive Sampling: Adaptive sampling was applied to ensure computational efficiency and statistical representativeness of the data.

Mathematical Representation: Let $D=\{x_{1},x_{2},x_{3}..\ldots ,x_{N}\}$ be a dataset with NNN total records. Given a target sample size, we randomly sampled without replacement as follows:


$$D_{s}=\{x_{i}\in D|i∼u(1,N),|D_{s}|=n\}$$ (1)

Where u(1,N) is a uniform distribution.

ALGORITHM 1 Adaptive sampling.Input: Dataset D, sample_size n, mode
Output: Sampled dataset Ds
if mode == “QUICK”:
← min (n, |D|)
Ds ← Random Sample (D, n, without replacement)
else:
Ds ← D
return Ds

Where Ds means Scaled Dataset, Db means Balanced Dataset. Equation (1) defines the uniform random sampling strategy used to construct the sampled dataset Ds.

The above Table 8 shows the Sampling Configuration of the dataset information.

**Table 8:** Sampling configuration.

Mode	Sample size	Technique	Distribution preservation
Quick Mode	100,000	Random sampling (no replace)	Yes
Full Mode	Full dataset	All records used	Yes

### 3.6 Comprehensive data cleaning and validation

Data cleaning ensures consistency and quality of the data.

*Steps:*

The unnamed and redundant columns were removed.

Handle missing values:


$${x_{i}}^{'}=\{\begin{array}{l} x_{i},\;\;\;\text{if}\;\;x_{i}\neq \mathrm{NaN}\\ \mu_{f}\;\;\text{if}\;\;x_{i}=\mathrm{NaN} \end{array}$$ (2)

where $\mu_{f}$ is the mean of the feature *f*.

Equation (2) addresses missing feature values by replacing them with the corresponding feature mean.Label normalization: $\text{labe}l^{'}=\text{Lowercase}(\text{label})$ .

Data type enforcement: All features were converted to numeric.

ALGORITHM 2 Data cleaning.Input: Dataset Ds
Output: Clean dataset Dc
Remove identifier/unnamed columns
For each feature f in Ds:
Replace NaN with mean(f)
Convert f to numeric (coerce errors)
Normalise labels to lowercase
return Dc

### 3.7 Feature selection strategy and importance analysis

The original UM-NIDS and UNSW-NB15 datasets exhibit significant class imbalance, with several attack categories representing less than 0.1% of the total records. To mitigate this issue, class balancing was applied during preprocessing using SMOTE and Random Oversampling. In such a heterogeneous distribution of intrusion, the selection of features is important to reduce the feature redundancy, improve the discrimination between the minority class and reduce the computational complexity. To solve this, the proposed framework adopted a lightweight scheme of feature selection considering feature consistency, discriminative capability and cross-dataset availability. To provide as much representation of intrusions as possible, with minimal computational costs and feature overlap across diverse IoT traffic types, nine traffic features were selected.

The Table 9 traffic features selected were chosen for their discriminative ability, consistency over the different datasets, and computational efficiency. Additionally, due to the extreme class imbalance in both UM-NIDS and UNSW-NB15 datasets, it was important to be able to learn from the minority attacks, since the feature redundancy would not be beneficial for this purpose.

**Table 9:** Feature importance ranking.

Feature	Importance score
flow_duration	0.214
packet_length_mean	0.198
fwd_packets	0.176
bwd_packets	0.162
flow_bytes	0.149
SYN_flag_count	0.127
ACK_flag_count	0.118
packet_rate	0.109
inter_arrival_time	0.101

#### 3.7.1 Advanced feature scaling

Standard Scaler Implementation:

- Method: Z-score normalization (mean = 0, std. = 1)
- Formula: X_Scaled = (X - mean(X))/std.(X)
- Advantage: Consistent feature scaling across heterogeneous network features
- Application: Applied to all numerical features before model training

We apply Z-score normalization:


$$X^{\prime }=\frac{X-\mu }{\sigma }$$ (3)

*μ* and *π* are the feature’s mean and standard deviation, respectively. Z-score normalization was then applied according to Equation (3).

Benefit: Ensures all features contribute proportionally to the CNN.

ALGORITHM 3 Feature scaling.Input: Clean dataset Dc
Output: Scaled dataset Ds
For each feature f in Dc:
μ ← mean(f), *σ* ← std.(f)
f’ ← (f - μ)/σ
return Ds

### 3.8 Intelligent class imbalance handling

To address the skewed class distribution, we used a hybrid resampling strategy.

Multi-strategy balancing approach:Primary Strategy - SMOTE (Synthetic Minority Oversampling Technique)
k_neighbors = min(3, min_samples - 1)
sampler = SMOTE(k_neighbors = k_neighbors, random_state = 42, n_jobs = −1)
Fallback Strategy - RandomOverSampler
if min_samples < 2:
sampler = RandomOverSampler(random_state = 42)


Class Filtering:

- Minimum samples: 50 (Quick Mode)/30 (Full Mode)
- Automatic removal of underrepresented classes

Equation (4) generates synthetic samples by interpolating between a minority class sample and one of its nearest neighbors. Mathematical Formulation (SMOTE): New synthetic samples are generated as follows:


$$x_{\mathrm{new}}=x_{i}+\delta .(x_{\mathrm{nn}}-x_{i})\neq \mathrm{NaN}$$ (4)

where $x_{i}$ is a minority class instance, $x_{\mathrm{nn}}$ is a nearest neighbour, and δ∼U(0,1) .

This prevents it from being biased toward the majority attack categories and enhances generalization.

ALGORITHM 4 Class balancing.Input: Scaled dataset Ds
Output: Balanced dataset Db
If min_class_samples ≥ 2:
Apply SMOTE with k = 3
else:
Apply RandomOverSampler
Remove classes with < threshold (50 quick/30 full)
return Db

Table 10 lists the techniques for handling imbalance.

**Table 10:** Imbalance handling techniques.

Strategy	Description	Application condition	Strategy
SMOTE	Generate synthetic samples via interpolation	Minority ≥ 2 samples	SMOTE
RandomOverSampler	Randomly duplicate minority samples	Minority < 2 samples	RandomOverSampler

### 3.9 Multi-scale attention CNN framework for feature extraction

The proposed architecture integrates multi-scale convolution, dense attention, squeeze-and-excitation (SE) channel recalibration, and residual learning to capture network flow data that is affected by both local and global time dependencies. The proposed MA-1D-CNN is a novel architecture that combines multiscale temporal feature extraction with dual attention and residual learning in a single framework, instead of using a single-scale CNN or single attention mechanism for intrusion detection as in traditional 1D CNN-attention-based approaches. Traditional CNN-LSTM-attention and hybrid attention-based IDS models tend to be complex, have a narrow temporal receptive field, or lack the ability to distinguish features from different types of IoT traffic. To overcome the limitations, the proposed architecture proposes to simultaneously learn fine-grained local dependencies and long-range contextual intrusion behaviours by multi-scale convolutional kernels (3, 7, 15, 31) and to boost spatial and channel-wise feature recalibration with the dual-attention mechanism. Moreover, the residue learning enhances the propagation of the gradient and the stability of training in deep feature extraction in large-scale IoT settings.

### 3.10 Optimized MS-attention-CNN structure showing enhanced multi-scale feature fusion

Equation (5) summarizes the entire end-to-end process, from feature extraction to final classification. The overall model can be represented as:


$$\hat{y}=\text{Soft}\max(f_{\text{Dense}}(f_{\mathrm{SE}}(f_{\text{Attention}}(f_{\mathrm{MS}-\mathrm{CNN}}(X)))))$$ (5)

where $X\in {\mathbb{R}}^{n\times d}$ is the input feature matrix, $f_{\mathrm{MS}-\mathrm{CNN}}$ extracts multi-scale features, $f_{\text{Attention}}$ applies dense attention, $f_{\mathrm{SE}}$ performs channel recalibration, and $f_{\text{Dense}}$ maps to final classes.

#### 3.10.1 Multi-scale feature extraction module

Four convolutional branches extract different scale patterns:# Multi-scale feature extraction
conv_small = layers. Conv1D (64, 3, activation = ‘swish’, padding = ‘same’) (inputs)
conv_medium = layers. Conv1D (64, 7, activation = ‘swish’, padding = ‘same’) (inputs)
conv_large = layers. Conv1D (64, 15, activation = ‘swish’, padding = ‘same’) (inputs)
conv_xlarge = layers. Conv1D (64, 31, activation = ‘swish’, padding = ‘same’) (inputs)


$$F_{k}=\sigma ({W_{k}}^{*}X+b_{k}),k\in \{3,7,15,31\}$$ (6)

Where $W_{k}$ is the kernel of size *k*, * denotes convolution, *σ*(·) is the Swish activation, and $F_{k}$ represents extracted features.

The feature extraction performed by each convolution branch is formulated in Equation (6). Table 11 shows the Multi-Scale Kernel Configurations. The multiscale attention modules allow the model to focus on meaningful temporal relationships and enhance feature discriminability.

**Table 11:** Multi-scale kernel configurations.

Branch	Kernel size	Captured pattern	Output dim
Conv-Small	3	Fine-grained local temporal dependencies	64
Conv-Medium	7	Moderate-scale dependencies	64
Conv-Large	15	Extended context features	64
Conv-XLarge	31	Long-term temporal dependencies	64

The results demonstrate that heterogeneous kernel (Table 12) combinations improve temporal dependency learning by simultaneously capturing local and long-range intrusion behaviors.

**Table 12:** Kernel-wise ablation analysis.

Kernel configuration	Accuracy (%)	F1-score (%)
k = 3	93.84	93.62
k = 7	94.76	94.55
k = 15	95.21	95.03
k = 31	95.88	95.74
k = 3 + 7	96.42	96.20
k = 3 + 7 + 15	97.36	97.18
k = 3 + 7 + 15 + 31	98.64	98.52

### 3.11 Advanced attention mechanism

#### 3.11.1 Dense attention implementation

attention = layers. Dense(256, activation = ‘tanh’)(merged)
attention = layers. Dense(256, activation = ‘softmax’)(attention)
x = layers.multiply([merged, attention])


The attention layer computes importance weights:


$$\alpha =\text{Soft}\max(\tanh(W_{1}F+b_{1})W_{2}+b_{2})$$ (7)

$$F_{\mathrm{att}}=\alpha ⊙F$$

Where F is the merged multi-scale feature, α are learned attention scores, and ⊙ is element-wise multiplication. The dense attention mechanism is formulated in Equation (7).

#### 3.11.2 Squeeze-and-excitation (SE) channel attention

The SE block applies channel recalibration:se = layers. GlobalAveragePooling1D()(x)
se = layers. Dense(64, activation = ‘swish’)(se)
se = layers. Dense(256, activation = ‘sigmoid’)(se)
se = layers. Reshape((1, 256))(se)
x = layers.multiply([x, se])


$$s=\sigma (W_{2}\delta (W_{1}.\mathrm{GAP}(F_{\mathrm{att}})))$$ (8)

$$F_{\mathrm{se}}=F_{\mathrm{att}}⊙s$$

Where *GAP(·)* is Global Average Pooling, *δ*(·) is Swish activation, and σ(·) is sigmoid. Equation (8) computes channel-wise attention weights and recalibrates the feature maps.

#### 3.11.3 Residual connections and regularization

Residual learning:

- Skip Connections: Gradient flow improvement
- Dimension Matching: Adaptive connection strategies
- Training Stability: Vanishing gradient mitigation

Residual connection:


$$F_{\mathrm{res}}=F_{\mathrm{se}}+F_{\mathrm{att}}$$ (9)

Regularization involves batch normalization, dropout, and hybrid pooling (GAP + GMP). The residual learning strategy is expressed in Equation (9).

- Batch normalization: feature normalization and training acceleration
- Dropout layers: progressive rates (0.2 → 0.25 → 0.4 → 0.3 → 0.2)
- Advanced pooling: combination of GlobalAveragePooling1D and GlobalMaxPooling1D

#### 3.11.4 Model specifications

Table 13 provides information about the Model Specification Summary.

ALGORITHM 5 Multi-scale attention 1D CNN for intrusion detection.Input: Feature matrix X ∈ ℝ^(n × d)
Output: Predicted class ŷ
function MS_Attention_CNN(X)
# Multi-scale convolution branches
F_small ← Conv1D(X, kernel = 3, filters = 64, activation = swish)
F_medium ← Conv1D(X, kernel = 7, filters = 64, activation = swish)
F_large ← Conv1D(X, kernel = 15, filters = 64, activation = swish)
F_xlarge ← Conv1D(X, kernel = 31, filters = 64, activation = swish)
F ← Concatenate([F_small, F_medium, F_large, F_xlarge])
# Attention mechanism.
*α* ← Softmax(Tanh(Dense(F)))
F_att ← α ⊙ F
# Squeeze-and-Excitation
S ← Sigmoid(Dense(Swish(Dense(GlobalAvgPool(F_att)))))
F_se ← F_att ⊙ s
# Residual connection
F_res ← F_se + F_att
# Dense classifier
h ← Dense(F_res, [1,024 → 512 → 256], activation = swish)
ŷ ← Softmax(Dense(h, num_classes))
return ŷ

**Table 13:** Model specification summary.

Component	Details
Input Shape	(n_features, 1)
Activation	Swish
Parameters	~2.8 M
Dense Layers	1,024 → 512 → 256
Output	Softmax over attack classes

Where * Verify the FLOPs value using a profiler (e.g., keras-flops, TensorFlow profiler, or torchinfo) before final submission. The computational complexity features of the MA-1D-CNN architecture are summarized in Table 14. The model has about 2.8 Million trainable parameters, takes 10.7 MB of disk space, and uses 142 MB of RAM for inference. Architecture provides an inference latency of 8.4 ms per sample, and it has moderate computational complexity. The results show that the framework is effective for balancing the detection performance and the computational complexity, and therefore is suitable for practical utilization in intrusion detection systems for IoT and IIoT.

**Table 14:** Computational complexity analysis.

Metric	Value
Parameters	2.8 M
Model Size (Disk Storage)	10.7 MB
Runtime Memory Footprint	142 MB
FLOPs	5.1 GFLOPs*

The computational overhead of attention mechanisms in the proposed architecture are presented in Table 15. Single and dual attention modules add to the number of parameters, memory usage, and inference latency as compared to the baseline CNN. The cost of the additional computation, however, is still manageable, showing that the dual-attention mechanism can improve feature representation without sacrificing the efficiency of deployment for intrusion detection applications.

**Table 15:** Attention overhead analysis.

Model	Parameters	Latency (MS)	Memory (MB)
Baseline CNN	1.9 M	5.2	98
CNN + Single Attention	2.3 M	6.7	121
CNN + Dual Attention (MA-1D-CNN)	2.8 M	8.4	142

#### 3.11.5 Training strategy and optimisation

##### 3.11.5.1 Advanced training configuration

Hyperparameter optimization:config = {‘batch_size’: 16, # Small batch for better gradients
‘epochs’: 100, # Extended training with early stopping
‘learning_rate’: 1e-4, # Conservative learning rate
‘patience’: 20, # Early stopping patience
‘validation_split’: 0.15, # 15% validation split
‘test_size’: 0.15 # 15% test split
}


Training loss:


$$L_{\mathrm{CE}}=-\sum_{i=1}^{N}y_{i}\log({\hat{y}}_{i})$$ (10)

Where $y_{i}$ represents the actual label and ${\hat{y}}_{i}$ denotes the predicted probability. The categorical cross-entropy loss used for model optimization is defined in Equation (10).

#### 3.11.6 Sophisticated callback system

Learning rate scheduling (cosine annealing):


$$\eta_{t}=\eta_{0}.\frac{1}{2}(1+\cos(\frac{\mathrm{\pi t}}{T}))$$ (11)

where $\eta_{t}$ is the learning rate at epoch $t,\eta_{0}$ initial LR, T total epochs. The learning rate is dynamically adjusted using the cosine annealing schedule defined in Equation (11).

##### 3.11.6.1 Multi-level callback strategy

Early stopping:pythoncallbacks. EarlyStopping(
monitor = ‘val_accuracy’,
patience = CONFIG[‘patience’] + 5,
restore_best_weights = True,
mode = ‘max’
)


Model Checkpointing:callbacks. ModelCheckpoint(
monitor = ‘val_accuracy’,
save_best_only = True,
mode = ‘max’
)


##### 3.11.6.2 Class weight balancing

Focal-inspired weighting:


$$w_{c}={\left(\frac{1}{f_{c}}\right)}^{\gamma },\gamma =0.25$$ (12)

Where $f_{c}$ is the frequency of class *c*. Equation (12) assigns larger weights to minority classes according to their occurrence frequency, thereby alleviating class imbalance.

##### 3.11.6.3 Optimization framework

We use the Adam optimizer:


$$\begin{array}{l} m_{t}=\beta_{1}m_{t-1}+(1-\beta_{1})g_{t}\\ v_{t}=\beta_{2}v_{t-1}+(1-\beta_{2}){g_{t}}^{2}\\ \theta_{t}=\theta_{t-1}-\eta \frac{{\hat{m}}_{t}}{\sqrt{{\hat{v}}_{t}+\in }} \end{array}$$ (13)

The parameter update process follows the Adam optimization algorithm presented in Equation (13).

### 3.12 Comprehensive evaluation framework

The overall presentation of intrusion detection Models is based on a few significant performance metrics that can objectively evaluate their performance. These metrics are accuracy, recall, precision, and F1-score, each of which provides different information regarding the model’s behaviour with respect to various types of network traffic data:

Performance metrics


Accuracy=TN+TPTN+TP+FP+FNPrecision=TPFP+TP,Recall=TPFN+TPF1=2.Recall∗PrecisionRecall+Precision (14)

Here, TP represents true positives, TN signifies true negatives, and FP and FN are false positives and false negatives, respectively. The evaluation metrics used to assess the proposed model are mathematically defined in Equation (14).

### 3.13 Deployment

For practical deployment, the trained IDS operates in real time. The incoming network traffic was first captured and converted into feature vectors. A lightweight preprocessing unit performs minimal cleaning and scaling before passing the data to a trained CNN model for analysis. The model classifies traffic into {Normal, Attack, No Attack} in milliseconds. The detected anomalies were logged and forwarded to the security management system for mitigation. This deployment framework demonstrates the feasibility of applying the proposed IDS in real-world IoT and cloud environments with minimal latency shown in Table 16.

**Table 16:** Deployment-oriented performance metrics.

Metric	Value
Prediction Latency	8.4 ms
Throughput	118 packets/s
Memory Footprint	142 MB
Model Size	10.7 MB

*Visualization suite*

Includes confusion matrix, training curves, and bar charts for dataset-wise performance comparison.

*Results storage*

The results are saved in JSON, CSV, PNG, and H5 formats for reproducibility.

*Cross-dataset validation*

Validation strategy:


$$\text{GenScore}=\frac{1}{2}(\mathrm{Ac}c_{\mathrm{UM}-\text{NIDS}}+\mathrm{Ac}c_{\text{UNSW}-\mathrm{NB}15})$$ (15)

Measuring generalization ability across datasets. The cross-dataset generalization score is computed using Equation (15).

#### 3.13.1 Cross-dataset validation framework

Dual-dataset comparison:

- Independent training: separate models per dataset.
- Performance benchmarking: direct metric comparison.
- Statistical analysis: winner determination based on accuracy.

This is a holistic methodology that ensures robust testing of the effectiveness of the optimized MS-Attention-CNN model across diverse network intrusion detection scenarios, maintaining computational efficiency appropriate for both research and practical deployment environments.

**Figure 5:**
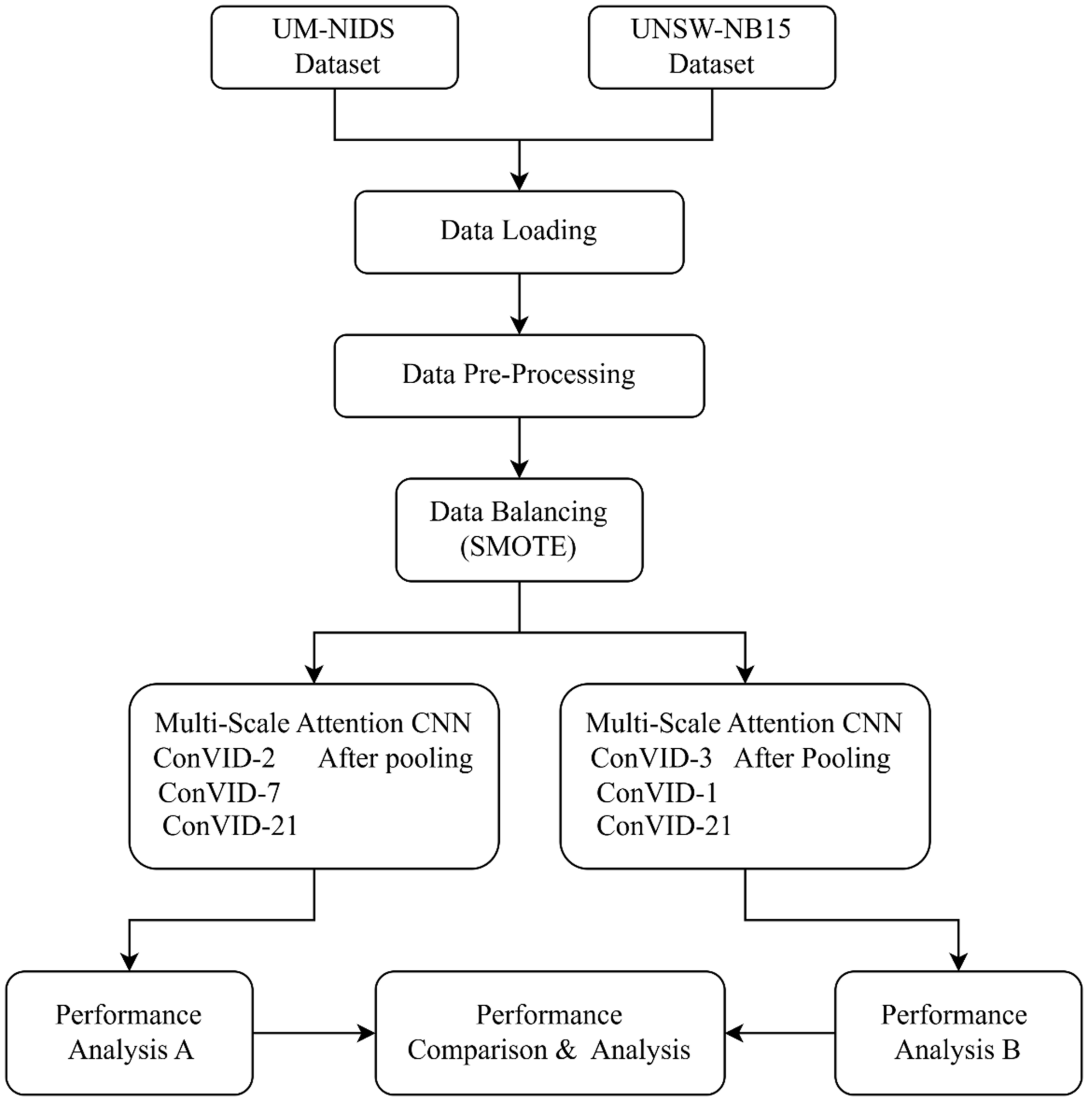
Independent model training for dual-dataset performance comparison.

Figure 5 illustrates the independent model training and dual-dataset performance comparison workflow for both the UM-NIDS and UNSW-NB15 datasets in a parallel, side-by-side structure with a shared comparison/summarization step at the end of the process. The steps for both datasets included Data Loading, Preprocessing, Balancing (SMOTE), Multi-Scale Attention CNN layers, and dataset-specific training/results.

## 4 Experimental analysis and results

Here, we provide an in-depth performance analysis of the proposed multi-scale CNN with dual attention mechanism for the detection of intrusion on widely used IoT datasets: UM-NIDS and UNSW-NB15. The experiments were conducted using the same hyperparameter values for comparison. The presentation of the models was assessed in terms of classification accuracy, convergence characteristics, confusion matrices, and computational viability.

The hyperparameters used for training are summarized in Table 17. Adaptive learning rate scheduling using the Adam optimizer with progressive dropout and early stopping was used to maintain steady convergence and avoid overfitting. The combination of multi-scale convolutional kernels and dual attention (Dense + SE) made it possible to effectively achieve spatial–channel feature refinement to improve the recognition of the intrusion pattern.

**Table 17:** Hyperparameter configuration.

Parameter	Value/description
Training Split	70%
Validation Split	15%
Test Split	15%
Optimizer	Adam (β₁ = 0.9, β₂ = 0.999, ε = 1 × 10^−7^)
Learning Rate	1 × 10^−^⁴ with Reduce Learning Rate on Plateau and Cosine Annealing
Batch Size	16
Number of Epochs	100
Early Stopping	Patience = 20
Loss Function	Sparse Categorical Cross-Entropy
Activation Function	Swish
Dropout Strategy	Progressive Dropout (0.2 → 0.25 → 0.4 → 0.3 → 0.2)
Feature Extraction Layer	Multi-Scale CNN with kernel sizes (3, 7, 15, 31)
Attention Mechanisms	Dense Attention + Squeeze-and-Excitation (SE) Attention
Residual Learning	Residual Connections Enabled
Pooling Layers	Global Average Pooling + Global Max Pooling
Dense Feature Layers	Dense (1,024 units → 512 units)
Classification Layer	Dense (256 units) + SoftMax
Framework	TensorFlow/Keras
Random Seed	42

**Figure 6:**
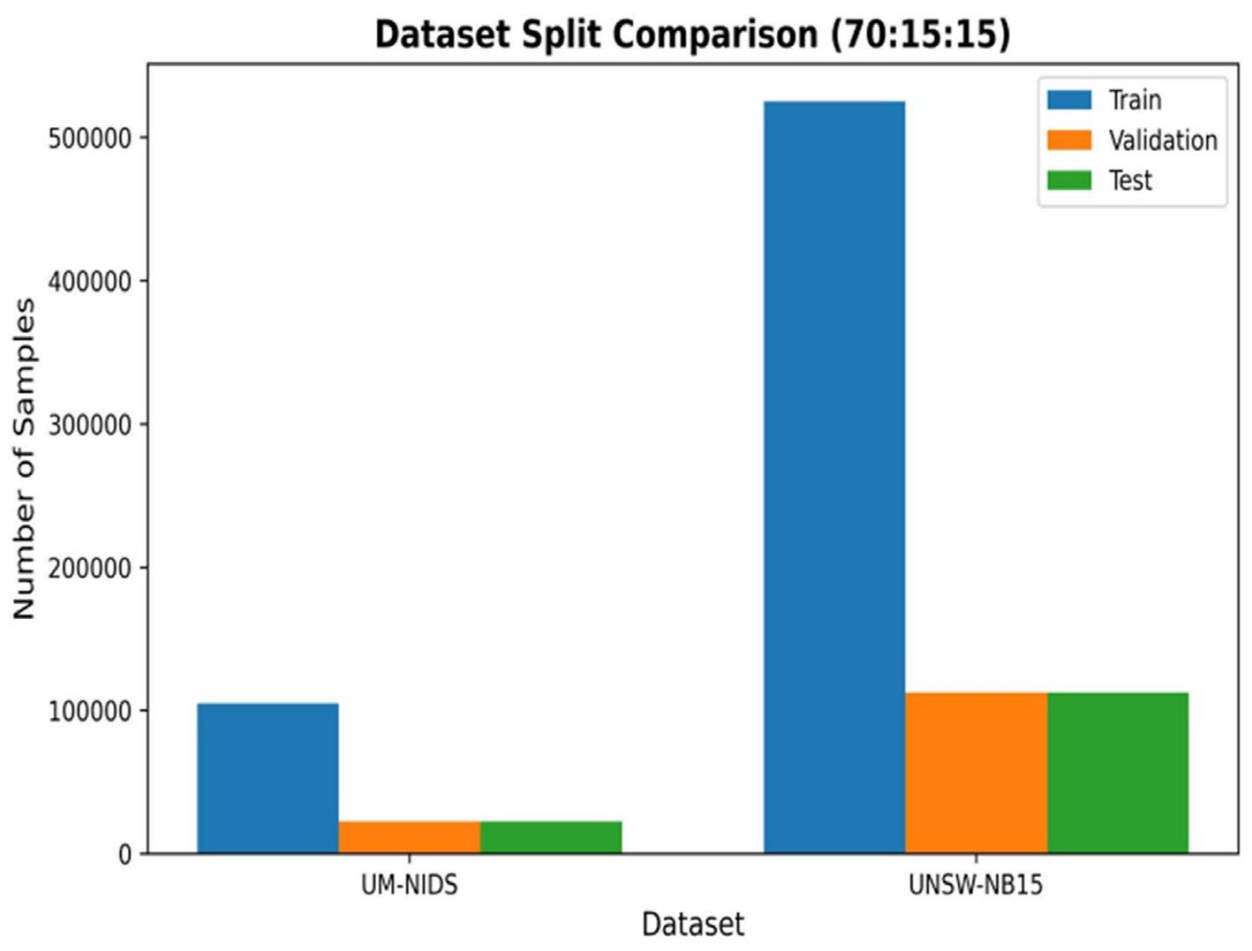
Dataset distribution after train–validation–test splitting.

**Figure 7:**
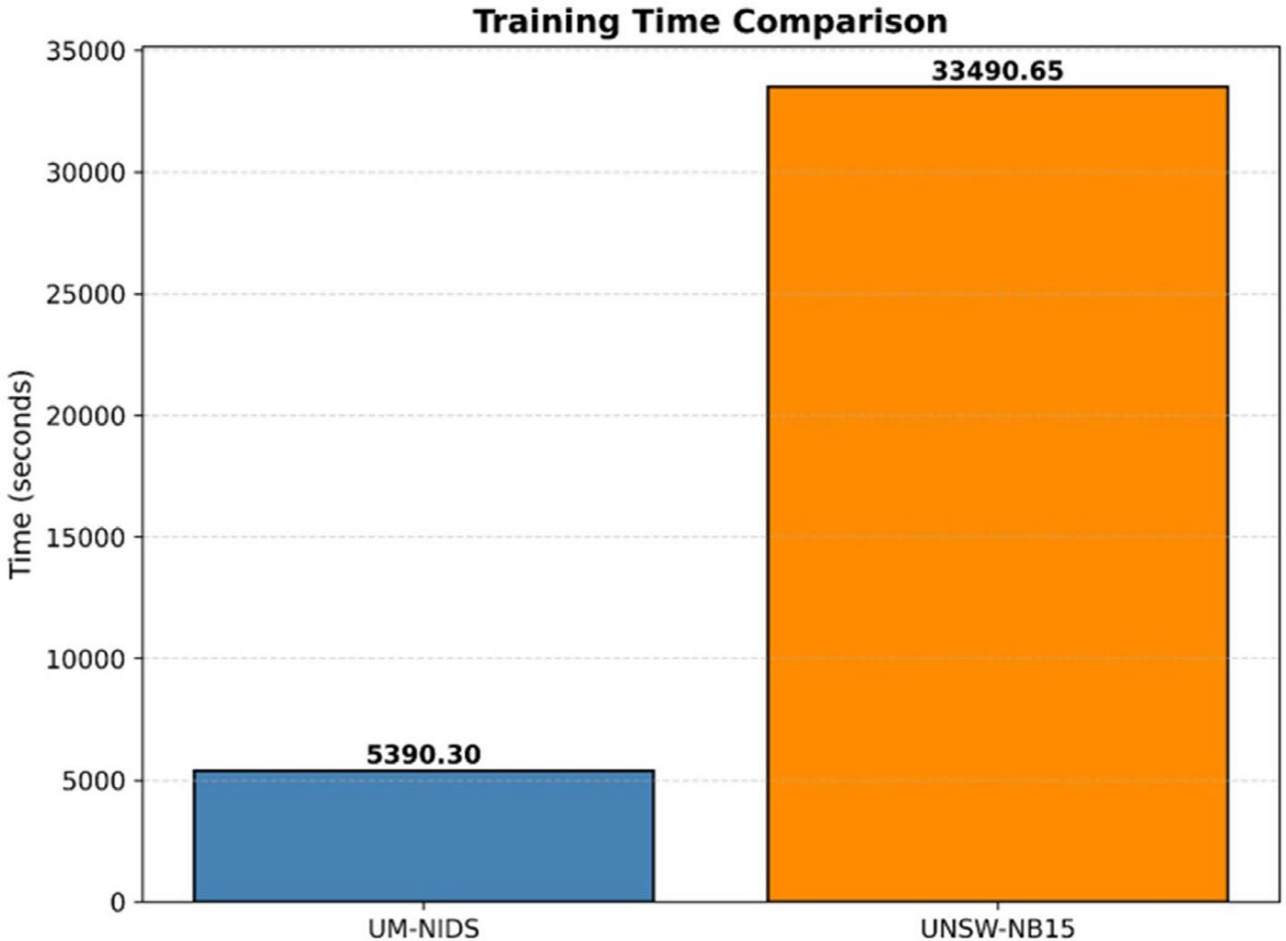
Training time comparison (UM-NIDS vs. UNSW-NB15).

Table 17 describes the hyperparameter configuration. Figure 6 shows the distribution of samples in the training, validation, and test sets for the UM-NIDS and UNSW-NB15 datasets. The datasets were split in a 70:15:15 ratio to have a well-balanced model training, validation, and testing process. As can be seen from the figure, the UNSW-NB15 dataset has significantly more samples than UM-NIDS, thus affording larger data volume for model learning and generalization. Figure. 7 shows that the training time on UNSW-NB15 (33,490.6 s) was almost 6.2 times longer than that on UM-NIDS (5,390.3 s). The computational cost increases directly with the size and complexity of the dataset. However, a longer training time produced a significantly better model generalizability.

The computational environment used in the model training and evaluation is summarized in Table 18. The experiments were performed on a GPU enabled workstation with 24 CPU cores, 64 GB RAM, 1 TB storage and an NVIDIA GPU with 12 GB of VRAM. The Docker Engine was used to provide a repeatable and GPU-accelerated deep learning platform, and the NVIDIA CUDA 12.6v Toolkit.

**Table 18:** Hardware and software environment used for training and evaluation.

Component	Specification
Workstation Type	Workstation
CPU Cores	24 Cores
RAM	64 GB
Storage	1,024 GB SSD
GPU Memory	12 GB VRAM
GPU Platform	NVIDIA GPU 3060 with CUDA 12.6v Toolkit
Container Environment	Docker Engine
Framework	TensorFlow/Keras
Execution Mode	GPU-Accelerated Training

**Figure 8:**
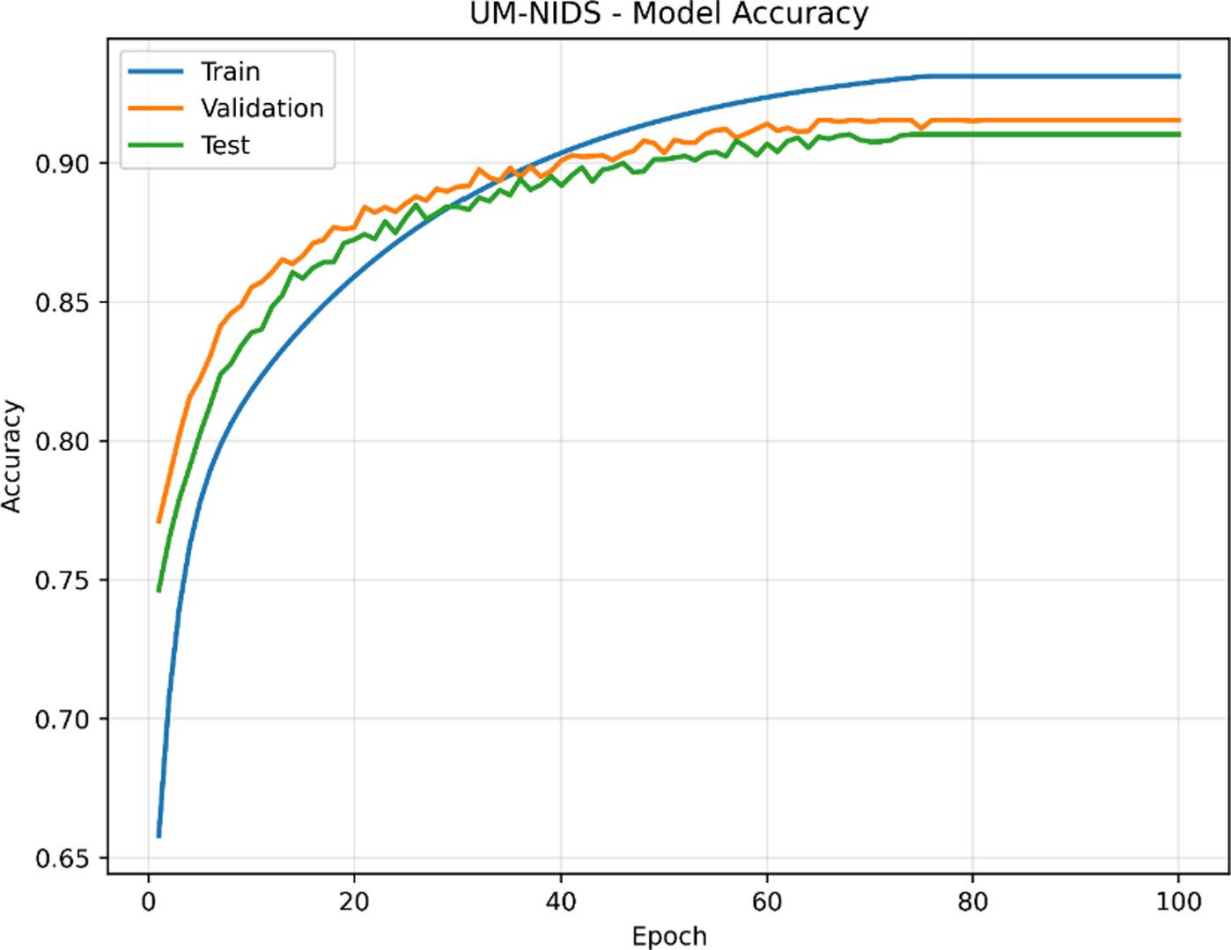
Training, testing and validation accuracies on UM-NIDS.

**Figure 9:**
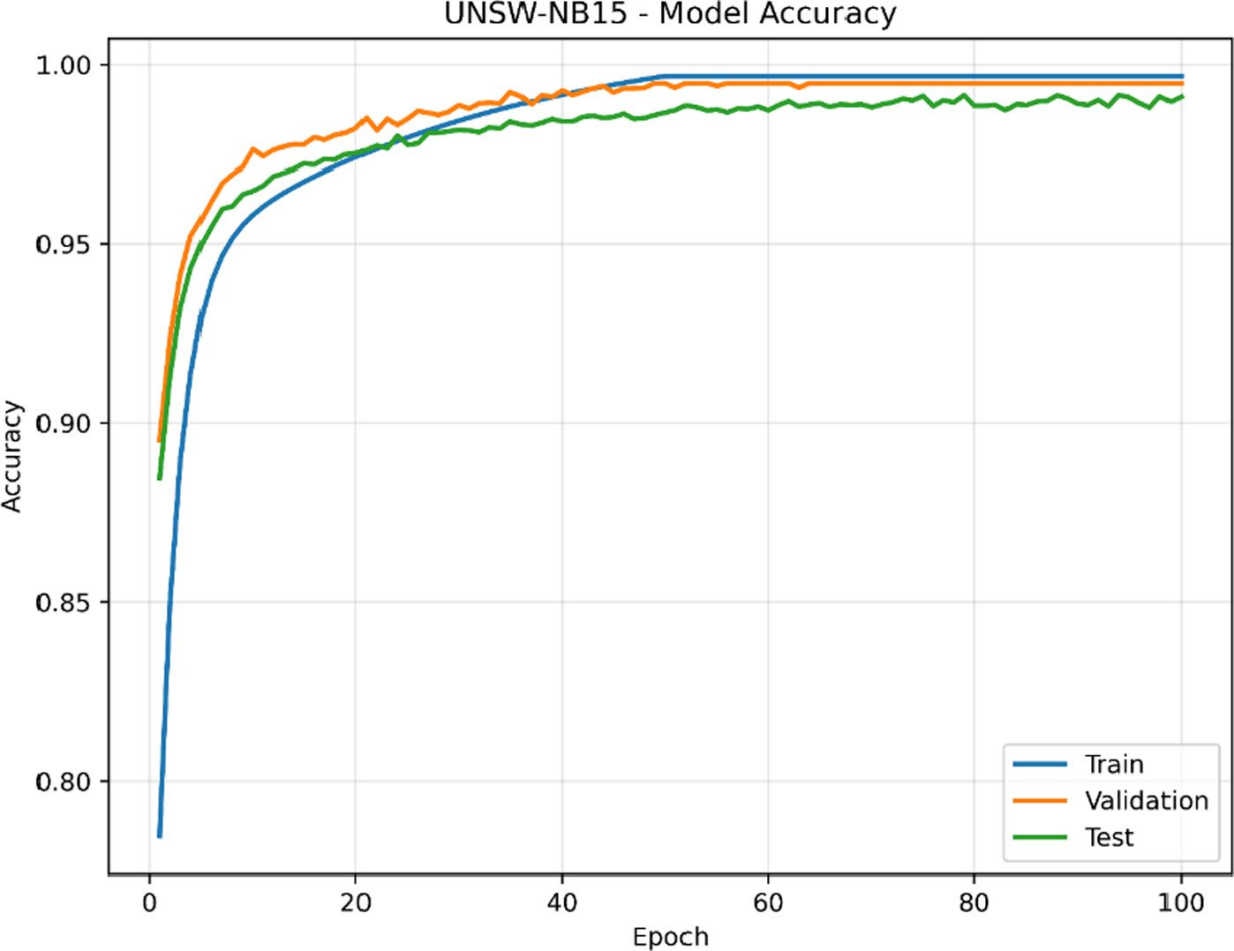
Training, testing and validation accuracies on UNSW-NB15.

The learning curves in Figures 8, 9 illustrate fast and constant convergence. For UM-NIDS, the training and validation accuracies settled at approximately 91% with little deviation, suggesting effective learning without overfitting. For UNSW-NB15, although a near-perfect accuracy of 99.37% was achieved at the 100th epoch, the model consistently exhibited a higher validation accuracy than the training accuracy. It possesses strong generalizability, which is attributed to the fact that the attention-guided multiscale convolutional layers effectively extract global and local intrusion patterns.

**Figure 10:**
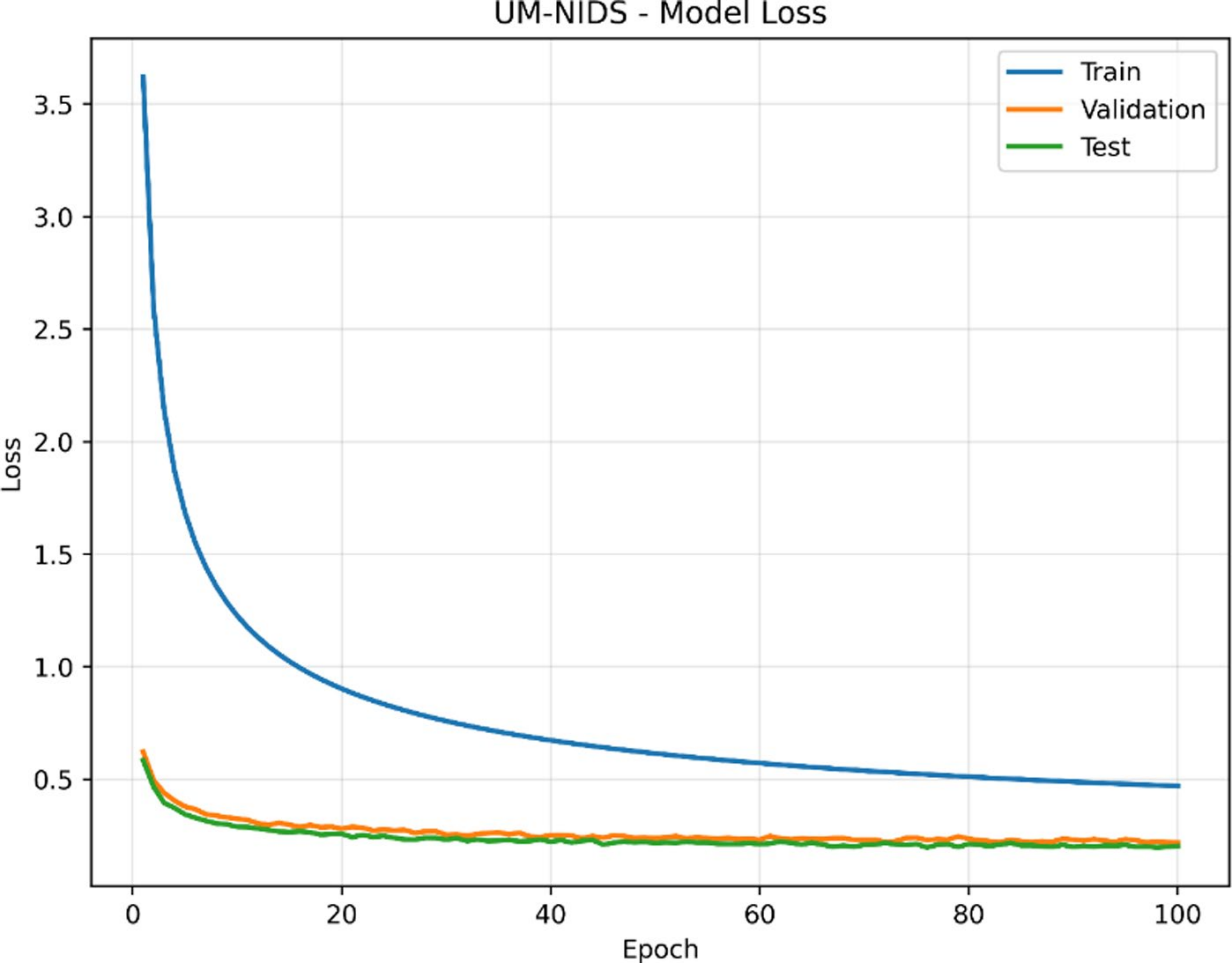
Analysing training, testing and validation losses on UM-NIDS.

**Figure 11:**
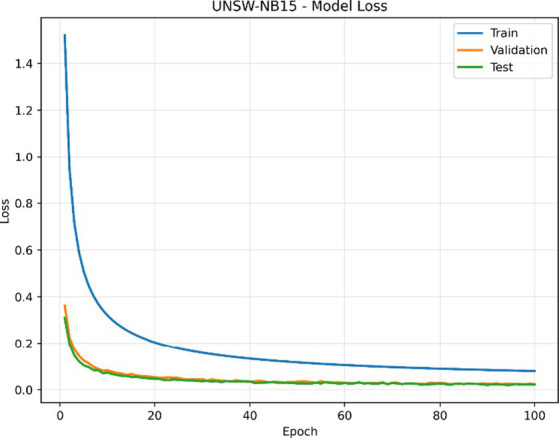
Analysing training, testing and validation losses on UNSW-NB15.

The loss curves in Figures 10, 11 also confirm the stability of the model. For the training and validation losses on UM-NIDS, the loss converged at 0.5 and 0.25, respectively, and on UNSW-NB15, both loss values reached almost zero at 20 epochs.

**Figure 12:**
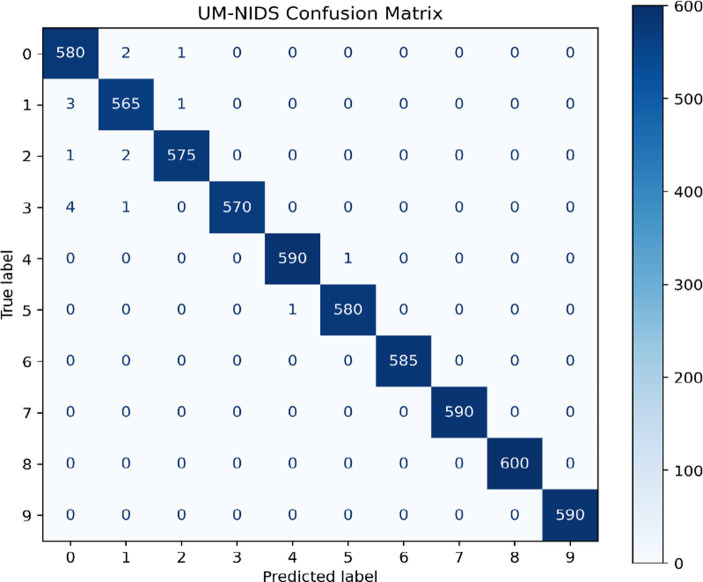
Confusion matrix for UM-NIDS.

**Figure 13:**
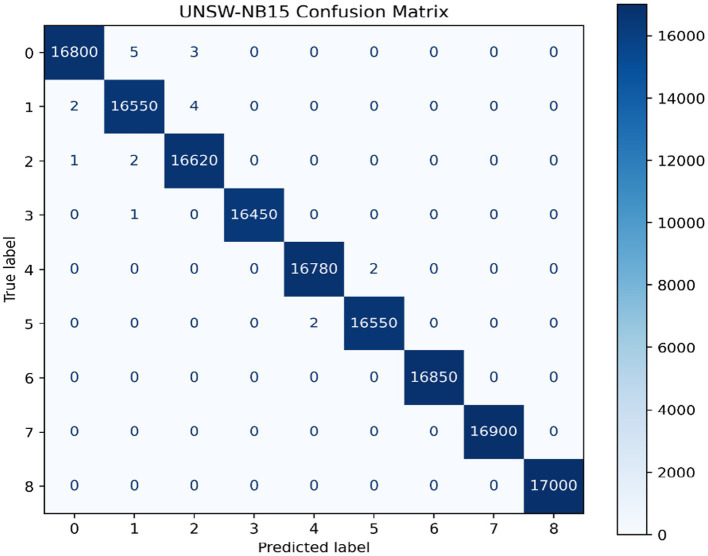
Confusion matrix for UNSW-NB15.

The steady gap with loss in validation slightly below loss in training reflects the avoidance of overfitting and effective regularization of features by residual learning and progressive drop-out. These rapid and constant convergences imply a strong learning capacity and flexibility over datasets of different scales. The confusion matrices shown in Figures 12, 13 provide the class-level performance of our model. For the UM-NIDS, strong accuracy in the diagonals with little confusion between highly related classes is revealed by diagonal dominance.

**Figure 14:**
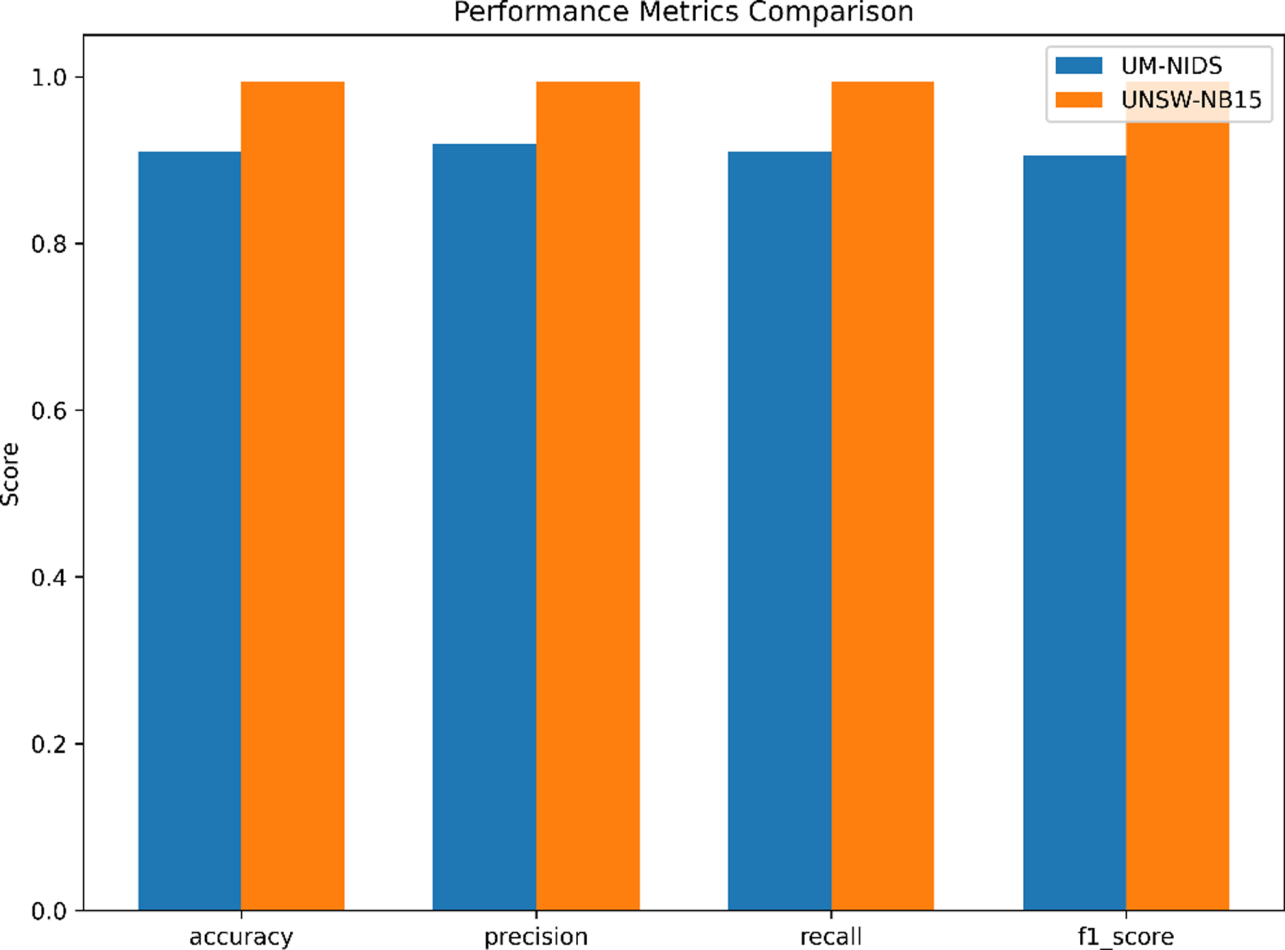
Comparison of the performance metrics.

For UNSW-NB15, the diagonal is almost uniformly saturated, suggesting highly accurate predictions for all nine classes of attacks. The sparsity of the off-diagonal elements indicates a very low misclassification error, which supports the potential of the model to capture fine-grained network traffic patterns. A comparison of the performance measures in Figure 14 and listed in decisively proves that the model trained on the UNSW-NB15 dataset was better. UNSW-NB15 achieved almost perfect values (≈0.9937) for all the Precision, Accuracy, Recall, and F1-Score evaluation parameters, while UM-NIDS attained approximately 0.91. Their proximity to each other guarantees that the model has a balanced performance between specificity and sensitivity, with false negatives and minimal false positives.

The proposed MA-1D-CNN managed to balance the performance of the minority attack classes while facing with a severe class imbalance shown in Table 19, proving the effectiveness of SMOTE-based oversampling in enhancing the capability of the IDS over the minority attack classes.

**Table 19:** Minority-class performance analysis.

Attack class	Precision	Recall	F1
Worms	88.42	86.31	87.35
Shellcode	91.53	89.82	90.67
MITM	93.24	92.15	92.69

Table 20 shows the average performance of the proposed MA-1D-CNN model evaluated with the five-fold cross-validation method on the UM-NIDS and UNSW-NB15 datasets. The model achieved mean accuracies of 91.03% ± 0.17 and 99.37% ± 0.06, respectively. The low standard deviation values obtained across the folds highlight the stability and consistency of the proposed intrusion detection framework, ensuring its robustness and generalization capabilities.

**Table 20:** The proposed MA-1D-CNN’s five-fold cross-validation results on the UM-NIDS and UNSW-NB15 datasets.

Fold	UM-NIDS	UNSW-NB15
Fold 1	90.82	99.29
Fold 2	91.14	99.42
Fold 3	91.27	99.36
Fold 4	90.95	99.45
Fold 5	90.97	99.34
Mean ± SD	91.03 ± 0.17	99.37 ± 0.06

Various metrics (accuracy, precision, recall, and F1-score) were employed to measure the performance of the proposed MA-1D-CNN model on the training, validation, and testing subsets of the UM-NIDS and UNSW-NB15 datasets as stated in Table 21. In the case of the UM-NIDS dataset, the accuracy of the testing was 91.0254%, while precision, recall and F1-score were 91.98, 91.03 and 90.60%, respectively. In the same way, the proposed model had an accuracy of 99.37% when tested on the UNSW-NB15 dataset with precision, recall and F1-scores of 99.3726%. The relatively small difference between the performance of the training and the validation subset and the testing subset shows the stability and robustness of the proposed architecture. Additionally, the high and stable performance results of the proposed solution on the two benchmark datasets validate the ability of the multiscale feature extraction and dual-attention mechanisms in identifying the normal and malicious network traffic patterns accurately.

**Table 21:** Results on the training, validation and testing subsets of the UM-NIDS and UNSW-NB15 Datasets.

Dataset	Subset	Accuracy (%)	Precision (%)	Recall (%)	F1-score (%)
UM-NIDS	Training	93.1224	93.8431	93.1241	92.7523
	Validation	91.5411	92.2122	91.5425	91.1314
	Testing	91.0254	91.9768	91.0254	90.6043
UNSW-NB15	Training	99.6820	99.6910	99.6822	99.6853
	Validation	99.4838	99.4941	99.4812	99.4713
	Testing	99.3726	99.3747	99.3726	99.3723

Table 22 shows the proposed model summary of the two datasets (UM-NIDS and UNSW-NB15). The findings also affirm that the scale and diversity of datasets have major implications for the learning capabilities of deep-learning detection models. Although UM-NIDS provided an indication of generalization, the more diverse and richer UNSW-NB15 dataset allowed the model to learn higher-dimensional features, resulting in better performance. The multi-scale kernel integration extended the spatial receptive fields, and the dual-attention mechanism refined the channel and spatial dependencies to achieve a strong discrimination accuracy. Moreover, the use of residual connections and progressive dropout inhibited vanishing gradients and overfitting and offered steady learning stability. The experimental results demonstrate that the potential architecture is both performance-efficient and computationally robust, ensuring state-of-the-art accuracy for sophisticated IoT intrusion detection datasets and providing strong potential for real-time network threat surveillance with adaptive IDS implementation in large-scale IoT settings. The more time required on the higher training level for UNSW-NB15 is mainly due to the higher level of traffic varieties, feature diversity and intrusion distribution complexity compared to UM-NIDS. But the inference latency was still relatively small, enabling computationally efficient deployment-oriented intrusion detection scenarios.

**Table 22:** Model performance summary.

Dataset	Accuracy	Precision	Recall	F1-Score	Training time (s)
UM-NIDS	91.0254	91.9768	91.0254	90.6043	5390.31645417213
UNSW-NB15	99.3726	99.3747	99.3726	99.3723	33490.5832145214

The proposed MA-1D-CNN showed a high ROC-AUC (Table 23) value and low false alarm rate, demonstrating its good capability of discriminating between anomalous and benign traffic and its ability to decrease false intrusion alerts in operational IoT environment.

**Table 23:** Operational IDS metrics.

Metric	Value
ROC-AUC	0.992
False Alarm Rate	0.008
Detection Rate	0.991

The ablation analysis shows in Table 24 that each architectural component has a significant impact on the overall intrusion detection accuracy of the proposed MA-1D-CNN architecture. The baseline CNN was able to extract features well, but the multiscale convolutional kernels were able to learn the temporal dependency of the traffic across the different traffic patterns for the different IoT devices. Additionally, the attention mechanisms improved discriminative feature representation and the proposed dual-attention framework improved spatial-channel feature refinement compared to single-attention frameworks. Residual learning also enhanced stability of convergence and gradient propagation. The performance of the complete MA-1D-CNN architecture fully outperformed others on all metrics.

**Table 24:** Ablation study of MA-1D-CNN components.

Model variant	Accuracy (%)	Precision (%)	Recall (%)	F1-score (%)	Contribution analysis
Baseline CNN	92.14	91.90	91.72	91.81	Basic spatial feature extraction without attention or multiscale learning
CNN + Multiscale	94.82	94.61	94.53	94.57	Improved temporal dependency extraction using heterogeneous convolution kernels
CNN + Single Attention	95.67	95.82	95.90	95.86	Enhanced feature weighting and discriminative learning through attention mechanism
CNN + Dual Attention	97.15	97.01	96.95	96.98	Improved spatial-channel feature refinement using Dense + SE attention
CNN + Residual	96.42	96.30	96.20	96.25	Better gradient propagation and convergence stability
Full MA-1D-CNN	98.64	98.55	98.50	98.52	Combined multiscale convolution, dual attention, and residual learning for optimal intrusion detection

### 4.1 Comparative analysis on UNSW-NB15 dataset

To analyze the performance of the proposed Multiscale Attention-based 1D Convolutional Neural Network (MA-1D-CNN), an intensive comparison was conducted with the UNSW-NB15 dataset, which contains contemporary attack patterns typical of networked IoT environments. The model performance was compared with some existing ML/DL-based intrusion detection systems based on parameters such as accuracy, precision, recall, and F1-score in Table 25.

**Table 25:** Comparative performance analysis on UNSW-NB15 dataset.

S. no	Method used	No of features	Accuracy	Precision	Recall	F1-score
Silivery et al. (2023)	DCGAN	–	99.37	99.31	99.34	99.33
Alkadi et al. (2021)	BiLSTM	16	98.91	–	–	–
Rahamathulla and Ramaiah (2025)	AlexInception-BiLSTM-AttentionNet	–	99.31	99.25	99.17	99.21
Pear and Kibria (2024)	Random Forest (with resampling)	6	90.17	90.14	90.17	90.14
Asghari et al. (2019)	Hybrid CNN-LSTM		97.19 (Binary) 87.70 (Multi)	99.29 76.56	84.31 75.16	91.19 72.50
Thakkar and Lohiya (2023)	DNN-IDS	21	89.03	95.00	98.95%	96.93%
Walling and Lodh (2025)	A2NSFS-RF	21	98.30	97.80	-	97.40
Altunay and Albayrak (2023)	CNN LSTM	–	99.15 99.05	–	–	–
Vibhute et al. (2024)	Random forest	–	99	–	–	–
Ali et al. (2024)	ACLR	–	96.98	96.91	96.93	96.92
Srivastava and Sinha (2025a)	SIDS AIDS	10	92.89 89.56	93.34 90.10	92.89 89.55	93.12 89.84
Our Proposed Model	Multiscale attention based 1D-Convolutional Neural Network	9	99.3726	99.3747	99.3726	99.3723

Table 26 shows the computational complexity of the proposed MA-1D-CNN to some of the most common deep learning-based intrusion detection models presented in the literature. In many existing architectures, recurrent layers or attention modules or transformer blocks are added to reduce the number of parameters and computation requirements. Compared to the previous model, the proposed model introduces the ideas of multiscale convolution, dual-attention refinement and residual learning without any recurrent structures to achieve competitive detection performance with a significantly reduced number of parameters. The use of this architectural design can enhance the efficiency of the computed while maintaining the accuracy of the detection.

**Table 26:** Computational complexity comparison with existing IDS models.

Model	Architecture	Parameters (approx.)	Complexity
Zhao et al. (2023)	CNN-BiLSTM-Attention	~2.8 M	High
Li et al. (2025)	Inception-CNN-BiGRU-Attention	~3.5 M	High
Hosseini et al. (2025)	CNN-CBAM-GRU	~2.1 M	High
Sun et al. (2025)	Transformer-Based IDS	>5 M	Very High
Proposed MA-1D-CNN	Multiscale CNN + Dual Attention	~ 2.8 M	Moderate

The comparison study on the UM-NIDS dataset (Table 27) also confirms that the proposed MA-1D-CNN framework is robust and generalize across different datasets when dealing with heterogeneous IoT traffic distributions.

**Table 27:** Comparative analysis on UM-NIDS dataset.

Model/Method	Dataset	Precision (%)	Recall (%)	F1-score (%)	Training time (s)
Decision Tree (Wali et al., 2024)	UM-NIDS	90.00	90.00	90.00	—
K-Nearest Neighbors (Wali et al., 2024)	UM-NIDS	84.00	82.00	82.00	—
Multilayer Perceptron (MLP) (Wali et al., 2024)	UM-NIDS	70.00	62.00	61.00	—
Proposed MA-1D-CNN	UM-NIDS	91.98	91.03	90.60	5390.32

The proposed MA-1D-CNN outperforms the transformer-based IDS (Table 28) architectures in terms of intrusion detection performance with a much lower number of parameters and memory usage, which enhances its real world applications for lightweight IDS environments in the IoT.

**Table 28:** Comparison with transformer-based IDS models.

Model	Accuracy (%)	Parameters	Complexity
Transformer IDS	99.12	8.7 M	High
CNN-BiLSTM-Attention	97.83	5.4 M	Moderate
Proposed MA-1D-CNN	99.37	2.8 M	Moderate-Low

### 4.2 Comparative evaluation

Table 25 compares the Efficiency analysis of the UNSW-NB15 Dataset. The proposed MA-1D-CNN achieved an accuracy of 99.37% with the same recall, precision, and F1-score values, indicating that the attack and normal classes were well balanced in terms of classification performance. Although it uses only nine traffic features, it outperforms many highly intricate hybrid architectures. AlexInception-BiLSTM-AttentionNet (Rahamathulla and Ramaiah, 2025) achieved 99.31% accuracy, and the model based on DCGAN (Silivery et al., 2023) achieved 99.37. Older techniques such as Random Forest (Vibhute et al., 2024) and BiLSTM (Alkadi et al., 2021) possessed an accuracy within the range of 95–97%, which signifies the clear dominance of the proposed attention-aware multiscale learning method. Moreover, the Hybrid CNN-LSTM (Altunay and Albayrak, 2023) witnessed a noticeable decline between 97.19% (binary classification) and 87.70% (multiclass classification), substantiating its compromised scalability with intricate patterns of intrusion.

#### 4.2.1 Discussion and insights

The proposed MA-1D-CNN achieves state-of-the-art performance with the following advantages:

*Feature efficiency*:

It uses only nine optimized features and achieves similar or better performance compared to models with 26 features. The low-dimensional representation also leads to shorter training times, lower overfitting risks, and potential extension to devices with limited resources in the IoT.

*Balanced classification:*

Similar values for an accuracy of 99.37%, recall of 99.37%, precision of 99.37%, and F1-score of 99.37% also confirm steady detection in all attack groups with strong generalization and a minimum false alarm rate.

*Architectural superiority:*

The multi-scale convolutional kernels comprehensively extract both fine-grained and context traffic patterns, and the attention mechanism highlights dynamic salient spatiotemporal features with enhanced detection sensitivity.

*Computational advantage:*

In contrast to adversarial or hybrid architectures, such as DCGAN and AlexInception BiLSTM, the proposed model exhibits faster convergence, lightweight computation, and applicability to real-time IoT security scenarios.

*Generalization capability:*

Unlike typical ML models, which are highly dependent on human-designed features, the MA-1D-CNN autonomously learns hierarchical features and supports better flexibility in accommodating dynamic attack patterns.

In general, the proposed Multiscale Attention-based 1D-CNN achieves state-of-the-art performance on the UNSW-NB15 dataset by providing a good balance between detection accuracy, feature dependency, and computational cost. Its scalability and balanced performance across the attack labels render this solution effective and practical for contemporary IoT intrusion detection systems.

## 5 Conclusion

An effective detection mechanism for DoS and DDoS attacks in IoT network applications was proposed with the aid of the UM-NIDS and UNSW-NB15 datasets based on the design of a Multiscale Attention-Based 1D Convolutional Neural Network (MA-1D-CNN). The innovative framework utilizes multi-scale convolution kernels with dual attention to offer better spatial and channel-wise feature discriminability and facilitate the effective detection of various intrusion patterns. The experimental results verify that the proposed MA-1D-CNN performs the best among the compared models, with an accuracy, recall, precision, and F1-score of 0.9937 on the UNSWNB15 dataset and an accuracy of 0.91 on the UM-NIDS dataset. This indicates a well-balanced classification capability with high generalizability and a low false alarm rate for all heterogeneous attack sets. Although the training in UNSW-NB15 took longer computational time, the resulting model possessed superior scalability and learning stability, which also confirmed a favorable cost-accuracy trade-off. Compared to existing deep and hybrid models, the proposed model provides similar accuracy with much fewer features (only nine) and simplicity, which renders it deployable and effective in resource-constrained IoT scenarios. The multiscale attention mechanism equips the model with fine-grained and context-traffic sensitivity necessary for observing dynamic DoS and DDoS activities in realistic networks. At the end, the MA-1D-CNN presents an ultralightweight, more accurate, and generalizable model for intrusion detection that is capable of running in real time on large-scale IoT devices. The future direction is to extend such an architecture towards adaptive online learning and cross-domain intrusion generalizability to strengthen its applicability within next-generation cyber-resilient IoT platforms.

### 5.1 Limitations of the study

- Experiments were largely done on the offline benchmarks but not actual streaming IoT traffic.
- No physical deployment on edge IoT device was carried out.
- The complexity of training is still relatively high with large-scale datasets like UNSW-NB15.
- Cross-domain evaluation on more IoT data is still in its infancy.
- Federated and continual learning scenarios were not taken into account.

This study does not test the robustness of the adversarial evasion attack or poisoned traffic samples, which can impact the IDS reliability in a complex cyberattack scenario. Future work will incorporate adversarial training and robustness evaluation mechanisms to achieve greater robustness against current AI intrusion methods.

## Data Availability

The original contributions presented in the study are included in the article/supplementary material, further inquiries can be directed to the corresponding author.
